# *Lactobacillus paracasei* Supplementation Prevents Early Life Stress-Induced Anxiety and Depressive-Like Behavior in Maternal Separation Model-Possible Involvement of Microbiota-Gut-Brain Axis in Differential Regulation of MicroRNA124a/132 and Glutamate Receptors

**DOI:** 10.3389/fnins.2021.719933

**Published:** 2021-08-31

**Authors:** Christopher Karen, Douglas J. H. Shyu, Koilmani Emmanuvel Rajan

**Affiliations:** ^1^Behavioural Neuroscience Laboratory, Department of Animal Science, School of Life Sciences, Bharathidasan University, Tiruchirappalli, India; ^2^Functional Genomics Laboratory, Department of Biological Science and Technology, National Pingtung University of Science and Technology, Neipu, Taiwan

**Keywords:** early-life social stress, maternal separation, probiotics, *Lactobacillus*, microbiota-gut-brain-axis, anxiety/depression-like behavior

## Abstract

This study was designed to investigate stressful social experience (SSE) in early life by examining how it can induce alterations in the microbiota-gut-brain axis. To test this, different experimental groups of pups experienced the presence of either a stranger (S) with mother (M+P+S) or without their mother (MS+S−M). Animals were assessed for anxiety-like behavior and high-throughput bacterial 16s rRNA sequencing was performed to analyze the structure of the gut microbiota. Our analysis revealed that early life SSE induced anxiety-like behavior and reduced the diversity and richness of gut microbiota. In the second experiment, all groups were supplemented with *Lactobacillus paracasei HT6*. The findings indicated that *Lactobacillus* supplementation had a significant beneficial effect on anxiety-like behavior in stressed rats (MS, M+P+S, and MS + S−M) accompanied by normalized levels of adrenocorticotropic hormone (ACTH), corticosterone (CORT), glucocorticoid receptor (GR), serotonin (5-HT), dopamine (DA), and noradrenaline (NA). Concomitantly, the expression of microRNA (miR)-124a was down-regulated and miR-132, caspase-3, glutamate receptors (GluR1, GluR 2; NR2A, and NR2B) were up-regulated in stressed groups but remained unchanged by *Lactobacillus* supplementation in stressed individuals. This indicates that stress-associated GluR1-GR altered interactions can be significantly prevented by *Lactobacillus* supplementation. Analysis of the fecal metabolite profile was undertaken to analyze the effect of *Lactobacillus*, revealing that five predicted neuroactive microbial metabolites were reduced by early life SSE. Our results showed a potential link between Lactobacillus supplementation and beneficial effects on anxiety-like behavior, the mechanism of which could be potentially mediated through stress hormones, neurotransmitters, and expression of miRNAs, glutamate receptors, and the microbiota-gut-brain axis.

## Introduction

Maternal separation (MS) has been established as a model to investigate early life stress (ELS) induced neurobiological and behavioral disorders later in life (Bian et al., 2015; Feifel et al., 2017; Wang et al., 2017). Previous studies reported that the impact of ELS on the hypothalamic-pituitary-adrenal (HPA) axis induces changes in adrenocorticotropic hormone (ACTH), corticosterone (CORT), glucocorticoid receptor (GR), neurotransmitters (Lai and Huang, 2011; Clarke et al., 2013; Chen and Baram, 2016), diversity of the gut microbiota (O’Mahony et al., 2011; Fukui et al., 2018) and associated molecules (Martisova et al., 2012; Clos-Garcia et al., 2019). Indeed, ELS highly influences the expression of microRNAs (miR) (small non-coding RNA molecules), which is implicated in several transcriptional, posttranscriptional, and epigenetic mechanisms (Dwivedi, 2011; Karen and Rajan, 2019), providing another level of control on target gene expression.

Earlier studies have shown that miR-124a is sensitive to stress and acts on GR to alter the level of expression (Vreugdenhil et al., 2009; Mannironi et al., 2013). Whereas miR-132 also responds to stress and positively influences postsynaptic proteins; thus, it activates post-synaptic α-amino-3-hydroxy-5-methyl-4-isoxazolepropionic acid (AMPA) (GluR1, GluR2) and *N*-methyl-D-aspartate (NMDA) (NR2A and NR2B) (Kawashima et al., 2010; Wanet et al., 2012). Recently, accumulating evidence has demonstrated the existence of bidirectional communication between the HPA axis and the gut microbiota, and linking anxiety and depressive behavior (Crumeyrolle-Arias et al., 2014; Vuong and Hsiao, 2017), in addition to neuroactive microbial metabolites (Bernal-Morales et al., 2017; Kotlega et al., 2020). Subsequent studies have indicated that supplementation with probiotics such as *Lactobacilli* positively influence the gut microbiota and restore the HPA axis (Bercik et al., 2012), attenuating emotional behavior and cognitive impairment against stress (Bravo et al., 2011; Liang et al., 2015; McVey Neufeld et al., 2019).

Our previous studies demonstrated that stressful social experience (SSE) in early life significantly impaired social behavior in later life by altering the core enzymes involved in methylation and its associated epigenetic changes in acetylation, methylation, and the expression of brain-derived neurotrophic factor (BDNF) in the amygdala (Karen and Rajan, 2019). In this study, we hypothesize that early life SSE can induce dysbiosis, which subsequently influences the stress hormones (ACTH and CORT), neurotransmitters [serotonin (5-hydroxytryptamine; 5-HT), dopamine (DA), noradrenaline (NA), expression of miRs (124a/132) and synaptic proteins (GluR1, GluR2)]. NR2A, NR2B), and later promote anxiety/depressive-like behavior. Furthermore, we hypothesize that supplementation with *Lactobacillus paracasei* HT6 could decrease anxiety and depressive behavior through the microbiota-gut-brain axis and restore associated alterations.

## Materials and Methods

### Subject Animals and Housing

Pregnant Wistar rats (*Rattus norvegicus*) were housed individually in a standard laboratory cage (43 cm × 27 cm × 15 cm) under standard laboratory conditions (26 ± 2°C temperature and 50–60% humidity in a 12 h light/dark cycle) with *ad libitum* water and food (chow pellets). The day of birth of the pups was noted as the postnatal day (PND) – 0. To avoid handling stress, bedding material was partially replaced every 2 days to minimally disrupt the nests and ensure the home cage odor. The experimental protocol and procedure used in this study were reviewed and approved (Ref. No. BDU/IAEC/P13/2019) by the Institutional Animal Ethical Committee (IAEC), Bharathidasan University, Tiruchirappalli, India. Following the guidelines of the Committee for the Purpose of Control and Supervision of Animal Experiments (CPCSEA), India. The experiments were designed to minimize the number of animals and their suffering.

### Experiment – I

#### The Stressful Social Experience Paradigm of Early Life (SSE)

The experiment started from PND – 2 and ended after behavioral tests on PND – 34. Four groups of animals were used. (1) The pups of the control group and their mother (Con, *n* = 6) were undisturbed up to PND-23, except during cleaning and general handling. (2) The MS (*n* = 6) group, for which MS was carried out for 3 h [09:00 – 12:00 h] from PND-5 to 10 by transfer of the mother and her pups to another cage with home cage bedding and then the mother was transferred back to the home cage immediately. SSE was provided by introducing the stranger (S) into the cage in the presence of the mother with pups (M+P+S, *n* = 6) (3) during the absence of the mother (MS+S−M, *n* = 6) (4). A specially designed cage (standard laboratory cage: 43 cm × 27 cm × 15 cm) was divided by wire mesh into two chambers, to prevent physical contact of the stranger (senescence male; 18 months old) with the mother and/or pups. Home cage bedding was used during exposure to strangers to avoid additional stress and modification of maternal behavior. One half of the specially designed cage with home cage bedding was used to place the experimental animals and the stranger was placed (10:00 – 11:00 h) in the other half of the cage during PND-5 to 10 (Karen and Rajan, 2019). Supplementary Figure 1 shows the detailed experimental timeline. Up to weaning day (PND-23), the dams of the control group and their pups were left undisturbed (Karen and Rajan, 2019).

### Experiment – II

Similar to experiment I, all four groups control + Pro (*n* = 6), MS + Pro (*n* = 6), M+P+S + Pro (*n* = 6), and MS+S−M + Pro (*n* = 6) were supplemented with *L. paracasei* HT6 (per orally, p.o. by oral gavage; from PND-2 to 16). Earlier studies have demonstrated that ELS-induced behavioral changes and that biochemical deficits can be ameliorated by specific live bacterial preparations (García-Ródenas et al., 2006; Bercik et al., 2012; Liao et al., 2019; Wei et al., 2019). *L. paracasei* reverses the negative imprinting of neonatal stress on both intestinal barrier function and growth. The effect of *L. paracasei* can alleviate ELS in the MS model. *Lactobacillus casei* was selected due to its psychotropic effects on the HPA axis and neurochemical changes in the brain (García-Ródenas et al., 2006; Liao et al., 2019). *L. paracasei* HT6 was obtained as a general gift from Professor Douglas J. H. Shyu of the National Pingtung University of Science and Technology, Taiwan. The strains were cultured in Lactobacillus MRS Agar (Cat. # GM641, HiMedia, India) using the spread plate method, and then a single colony of *L. paracasei* was retrieved and inoculated in Lactobacillus MRS broth (Cat. # GM369, HiMedia, India) and cultured at 37°C for 18 h. The present study used an *L. paracasei* culture of 10^9^ cells of CFU as the fixed dose [starting from 20 μl on PND-2 to 100 μl on PND-16 with an incremental increase in volume of 20 μl for every 3 days] (Wei et al., 2019).

### Behavioral Test

#### Open Field Test (OFT)

On PND-33, OFT was performed to measure exploratory activity and anxiety-related behaviors. The test was conducted in a square arena (100 cm × 100 cm) divided into 25 equal squares with a white floor to evaluate the behavior in the new environment (Crawley, 1985). The experimental rat was placed individually in the corner of the behavioral arena facing the center and behavior was recorded for 5 min under bright light (550 lux). Their behavioral profiles [number of squares crossed, time spent at the central square, and number of entries to center square] were analyzed. The apparatus was cleaned with 75% ethanol after every behavior test.

#### Tail Suspension Test (TST)

Tail suspension test was conducted in PND-34 to assess depression-related behavior (Steru et al., 1985). Individuals were suspended 50 cm above the ground by the tail with an adhesive tape (Leukoplast^®^) and their immobility and movement behaviors were recorded for 6 min, except for the whiskers and respiration, movement was measured as the index of despair. To prevent tail climbing behavior, a polycarbonate tube was placed around the tail of each rat immediately before the test (Lad et al., 2007).

### Hormonal Assay

Blood samples were collected in a tube with anticoagulant (sodium citrate: 0.5 ml of 3.8% solution per 4.5 ml of blood) from individuals in the experimental group, blood plasma was separated by centrifuging at 1,800 rpm for 10 min (Mohri and Rezapoor, 2009) and stored at −20°C for the estimation of hormones and neurotransmitters. The level of CORT and ACTH was estimated (ELISA kit, ALPCO Diagnostics, Salem, NH). The whole brain was dissected out, placed on ice, and then the prefrontal cortex (PFC) was removed (Heffner et al., 1980). The obtained PFC tissue (25 mg) was washed and then homogenized in the supplied buffer (50 μl), the supernatant was collected by centrifuging (10,000 rpm at 4°C) for 10 min and stored at −80°C. The level of PFC CORT was estimated using an ELISA kit (ALPCO Diagnostics, Salem, NH).

### Neurotransmitter Analysis

Blood plasma samples were used to estimate the level of 5-HT. PFC tissues were homogenized in a buffer (perchloric acid, disodium ethylenediaminetetraacetate dihydrate, and reduced glutathione), centrifuged at 12,000 rpm for 20 min at 4°C, and their supernatant was stored at −80°C. The level of 5-HT, DA, and NA were determined using a 5-HT EIA kit (BioSource Europe S.A., Belgium), DA EIA kit (R&D Systems, MN, United States), and NA kit (Immuno-biological Laboratories Inc., Minneapolis, MN, United States) using the manufacturer’s instructions.

### Analysis of Fecal Microbiota Composition by 16S rRNA Gene Sequencing

In PND-27, a sterile plastic sheet was placed in half of the home cage. The moist fecal boli that were collected on top of the plastic sheet were collected in screw cap vials without DNase and RNase. After collection, samples were immediately frozen and stored at −80°C and used to examine the microbial diversity and the profile of fecal metabolites with gas chromatography and mass spectrometry (GC/MS) analysis. Bacterial DNA for sequencing was isolated from stool according to the manufacturer’s instructions (FavorPrep^TM^ stool DNA plus isolation mini kit; Cat. # FAPST 001; FAVORGEN Biotech Corporation). The DNA samples were estimated using Biophotometer plus (Eppendorf Inc., Germany).

Specific primers (16S V3–V4 341F: 5′-CCTACGGGNGGCWGCAG-3′ and 805R: 5′-GACTACHVGGGTATCTAATCC-3′) (Sinclair et al., 2015) were used to amplify the specific 16S rRNA region with KAPA High-Fidelity PCR Master Mix (KAPA^®^ Biosystems). The reaction conditions were initial denaturation at 95°C for 3 min, following denaturation at 95°C for 30 s, annealing at 57°C for 30 s, extension at 72°C for 30 s, for 30 cycles, and final extension at 72°C for 5 min. The PCR products were then examined in a 2% agarose gel and then purified with the QIAquick gel extraction kit (Qiagen). Sequencing libraries were generated using the Truseq nano DNA library prep kit (Illumina, United States) following the manufacturer’s instructions and index codes were added. Library quality was evaluated on the Qubit@ 2.0 fluorometer (Thermo Fisher Scientific Inc.) and the Agilent Bioanalyzer 2100 system (Agilent Technologies, Inc.). Finally, the library was sequenced on an Illumina MiSeq platform, generating 300 bp paired-end reads, and processed using quantitative insights into microbial ecology (QIIME) (Caporaso et al., 2010).

### Total RNA and Protein Isolation

The animals (*n* = 6) representing each group were sacrificed after the behavioral test. The whole brain was dissected and placed in an ice-cold Petri dish. The amygdala tissue was carefully dissected from the brain slice, as previously reported (Zapala et al., 2005). One portion of the dissected amygdale was used to isolate RNA and the other side was used to isolate the protein. Total RNA was isolated from amygdala tissue samples using PureZOL (Bio-Rad Laboratories Inc.; Cat. # 7326890), according to the manufacturer’s instructions. The RNA samples were estimated using Biophotometer plus (Eppendorf Inc., Germany). Total RNA (2 μg) was reverse transcribed into cDNA using random/oligo-dT primers (Bio-Rad Laboratories Inc.; iScript^TM^ cDNA synthesis kit; Cat. # 170-8891) and stored at 4°C.

Total protein was isolated by homogenizing amygdala tissue with ice-cold lysis buffer [Tris-Hydrochloric acid (Tris-HCl) pH 7.5, sodium chloride (NaCl), ethylenediaminetetraacetic acid (EDTA), dithiothreitol (DTT), tergitol (NP-40), sodium orthovanadate (Na_3_VO_4_), Phenylmethylsulfonyl fluoride (PMSF) and protease inhibitor cocktail (Cat # P8340; Sigma-Aldrich)]. The homogenates were incubated on ice for 30 min and then centrifuged at 12,000 rpm for 30 min at 4°C. The clear supernatant was collected in a fresh tube, centrifuged again at 12,000 rpm for 30 min at 4°C, and supernatants were stored at −80°C.

### Quantitative Real-Time PCR (qRT-PCR)

The qRT-PCR was performed in the CFX-96 Touch^TM^ Real-time PCR Detection System (Bio-Rad Laboratories) using the real-time reaction mixture (iQ^TM^ SYBR^®^ Green supermix, Bio-Rad Laboratories) according to the manufacturer’s instructions along with specific primers (100 pM) (miR-132 forward: 5′-GCAACCGTGGCTTTCGATTG-3′, reverse: 5′-GGCGACCATGGC TGTAGA-3′; miR-124a forward: 5′-TCCGTGTTCACAGCGGAC-3′, reverse: 5′-CATTCA CCGCGTGCCTTA-3′; U6SnRNA forward: 5′-CTCGCTTCGGCAGCACA-3′, reverse: 5′-AACGCTTCACGAATTTGCGT-3′) and cDNA (1.2 μg). Amplified with the following conditions: initial denaturation at 94°C for 30 s, denaturation at 94°C for 5 s, annealing (miR132 – 65°C; miR124a – 62°C; U6Sn RNA – 55°C) for 5 s and extension at 72°C for 5 s. The amplification of the single PCR product was confirmed by monitoring the dissociation curve followed by melt curve analysis. The U6SnRNA level was used as a housekeeping gene to normalize the relative expression levels of the target miR-132 and miR-124a. Each reaction was performed in triplicate. The PCR products were visualized in native polyacrylamide gel electrophoresis (12%) stained with ethidium bromide. Data were presented as mean fold change relative to the control group.

### Western Blot

An equal concentration of total proteins (40 μg) was resolved on 10% polyacrylamide gel. The separated proteins were transferred electrophoretically to a polyvinylidene difluoride (PVDF) membrane (*Trans*-Blot^®^ Transfer pack Cat # 1704156) using a *Trans*-Blot^®^ Turbo^TM^ Transfer System (Bio-Rad Laboratories). The membranes were blocked in Tris buffered saline (TBS) containing 0.1% Tween-20; 5% non-fat milk for 2 h at room temperature. The membrane was then incubated at 4°C for 8 h with one of the following specific primary antibodies [anti-GR (1:1000, Cat. # SC-1004; Santa Cruz Biotech)/anti-GluR-1 (1:2000, Cat. # ABP51437; Abbkine)/anti-GluR-2 (1:2000, Cat. # ABP51438; Abbkine)/anti-NR2A (1:2000, Cat. # BT-AP02388; Bio Assay Technology Laboratory)/anti-NR2B (1:2000, Cat. # BT-AP02389; Bio Assay Technology Laboratory)/anti-β-actin (1:1000, Cat. # SC-47778; Santa-Cruz Biotech)]. Membrane-bound antibodies were detected by incubation for 3 h using alkaline phosphatase conjugated secondary antibody [goat anti-rabbit IgG – ALP (1:2000, Cat. # 110018001A, GeNei^TM^)/goat antimouse IgG – ALP (1:2000, Cat. # 105215, GeNei^TM^)]. Subsequently, alkaline phosphatase activity was detected with 5-bromo-4-chloro-3-indolyl phosphate disodium salt (BCIP)/nitroblue tetrazolium chloride (NBT) (Merck, Cat. # ES006) according to the manufacturer’s instructions. The images were acquired with a ChemiDoc XRS molecular imager (Bio-Rad Laboratories) and the trace quantity of each band was measured using Image Lab 2 software (Bio-Rad Laboratories). Full Western blot (uncropped) image for all markers is shown in the Supplementary Information as Supplementary Figures 4–7. Data were presented as relative expressions to the control.

### Co-immunoprecipitation (Co-IP)

Spin columns were packed with AminoLink Plus Coupling resin slurry (50 μl) and washed with buffer (Cat. # 26149, Pierce Co-Immunoprecipitation Kit, Thermo Fisher Scientific Inc., IL, United States) then 10 μg of anti-rabbit polyclonal GR antibody (Cat. # SC-1004; Santa Cruz Biotech, 1:1000 adjusted to 200 μl volume with 1× coupling buffer) was added to the column and incubated on a rotator for 2 h at room temperature to immobilize antibodies. For Co-IP, 80 μg of total protein lysates from each experimental group (Con/MS/M+P+S/MS+S−M/Con+Pro/MS+Pro/M+P+S+ Pro/MS+S−M+Pro) were immunoprecipitated with GR antibody in immobilized spin columns overnight at 4°C. Immunoprecipitated proteins were eluted using elution buffer, and then a co-immunoprecipitation experiment was performed following the manufacturer’s instructions. An equal volume of GR precipitated protein representing each experimental group was analyzed by immunoblotting (as mentioned earlier) using anti-rabbit polyclonal GluR1 antibody (1:2000, Cat. # ABP51437; Abbkine).

### Gas Chromatography/Mass Spectrometry (GC/MS)

#### GC/MS Sample Collection

Feces (40 ± 5 mg) were homogenized in 800 μL of solvent (chloroform/methanol/water; v/v/v; 2:5:2 in ice water) and centrifuged at 12,000 rpm at 4°C for 15 min. The supernatant (100 μl) was mixed with pentadecane (5 μl) and pyridine (5 μl) in GC vials. The cells were pre-chilled at −80°C for 1 h and lyophilized under vacuum pressure (5 *Pa*) at −60°C for 1 h. Metabolites were obtained by vigorously vortexing the sample with methoxylamine hydrochloride in anhydrous pyridine (30 μl of 20 mg/ml). Subsequently, the vials were incubated at 37°C for 90 min and then 30 μl of bis-(trimethylsilyl) trifluoroacetamide regent in 1% trimethylchlorosilane (BSTFA in 1% TMCS) was added to the mixture and then incubated at 70°C for 60 min. Finally, 500 μl of methanol was added and gently mixed.

#### GC/MS Analysis

GC/MS analysis was performed using an SH-Rxi-5 Sil MS column (30 m × 0.25 mm × 0.25 μm) with Helium as carrier gas, the inlet purge flow was 3.0 ml/min and the column gas flow rate was 1 ml/min with Shimadzu gas chromatography mass spectrometry system (Shimadzu PTE LTD, Asia Pacific, Singapore, Cat. # GCMS-QP2020). Samples (1 μl) were injected in split mode, with an initial oven temperature at 70°C for 2 min, increased to 160°C with 6°C/min, then 240°C with 10°C/min, and finally increased to 300°C with 20°C/min for 6 min. The temperatures of injector, transfer line, and electron impact on ion source were set to 250, 290, and 230°C.

#### Metabolic Profile Analysis

The extraction, alignment, and further processing of raw GC/MS data were carried out using GC/MS post-run analysis software (GC/MS solutions version 4.45, Lab solutions).

### Statistical Analysis

Data were presented as mean ± standard error of the mean (SEM) and plotted with KyPlot (ver 1.0) for graphical representation. Two-way analysis of variance (ANOVA; Sigma Stat, ver 3.1) was used to examine the behavioral analysis between groups; then the Bonferroni *post hoc* test was performed. One-way analysis of variance (ANOVA) was used to examine the significant difference between groups in molecular data. Then, *post hoc* comparisons were performed with the Bonferroni test. Data were shown as mean ± SEM and ^∗^ indicates significant difference between groups (^∗∗∗^*P* < 0.001, ^∗∗^*P* < 0.01, ^∗^*P* < 0.05).

## Results

### Effect of Early-Life SSE on the Dynamics of the Intestinal Microbial Ecosystem

**FIGURE 1:**
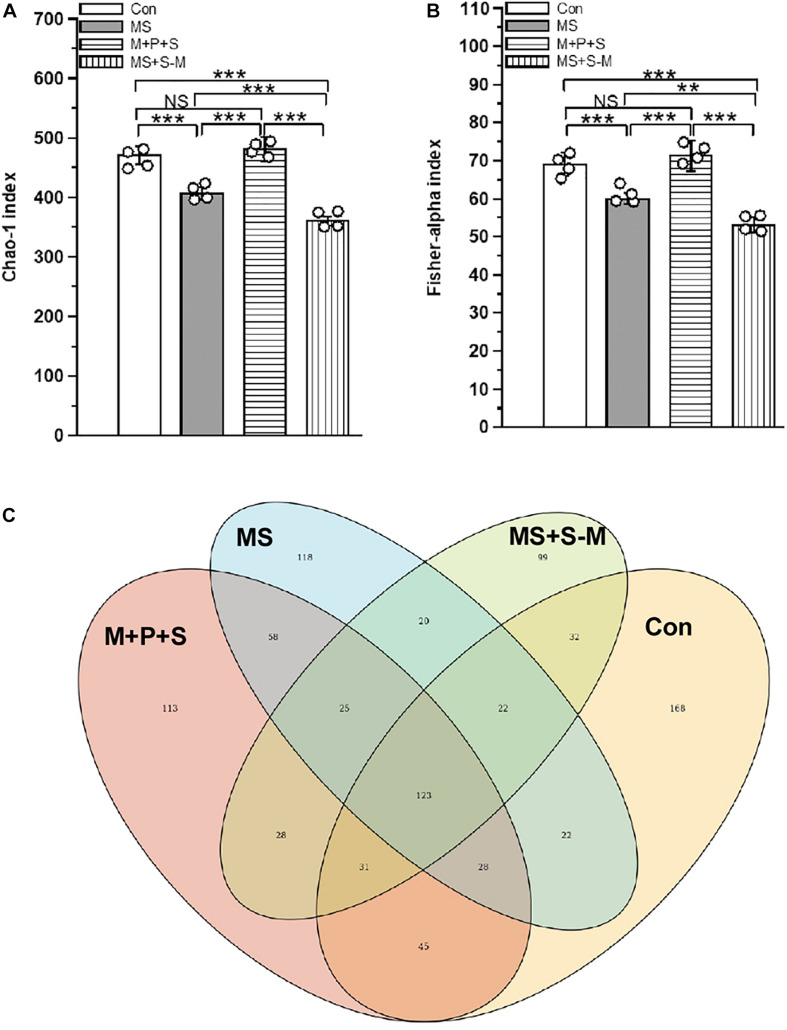
Alpha-diversity of the fecal microbiota in rat pups after experiencing early life social stress **(A)** Chao-1 index and **(B)** Fisher-alpha index. **(C)** Venn diagram showing the identified current major total bacterial OTUs at the genus level, and the overlapping area are listed with shared OTUs. Values are presented as mean ± SEM. Significant differences between groups are indicated by ***P < 0.001, **P < 0.01, NS, not significant.

Early life SSE altered the fecal microbiota compared to the control group. There was a significant difference between experimental groups in α-diversity determined by the Chao-1 index [*F*_(3,15)_ = 88.26, *P* < 0.001] and the Fisher alpha index [*F*_(3,15)_ = 152.900, *P* < 0.001] (Figures 1A,B and Supplementary Figure 2). *Post hoc* comparison indicated that microbial diversity in the MS (*P* < 0.001) and MS+S−M (*P* < 0.001) groups was significantly lower compared to the control and M+P+S groups. Compared to MS + S−M, a significantly lower diversity was estimated in the MS group (*P* < 0.001) than in the MS group, but there were no significant differences between the control and the M + P + S group (*P* = 0.932). As can be seen from the Venn diagram (Figure 1C) showing the identified operational taxonomic units (OTUs) in experimental groups, all experimental groups shared 14.59% (123) OTUs out of 843 OTUs, and the number of unique OTUs in the stressed group was lower in MS (13.99%/118 OTUs), M+P+S (13.4%/113 OTUs), and MS+S−M (11.7%/99 OTUs) compared to the control (19.9%/168 OTUs). When we compare the stressed groups, MS+S−M shared 20 OTUs (27.39%) with the MS group and 28 OTUs (38.35%) with the M + P + S groups.

**FIGURE 2:**
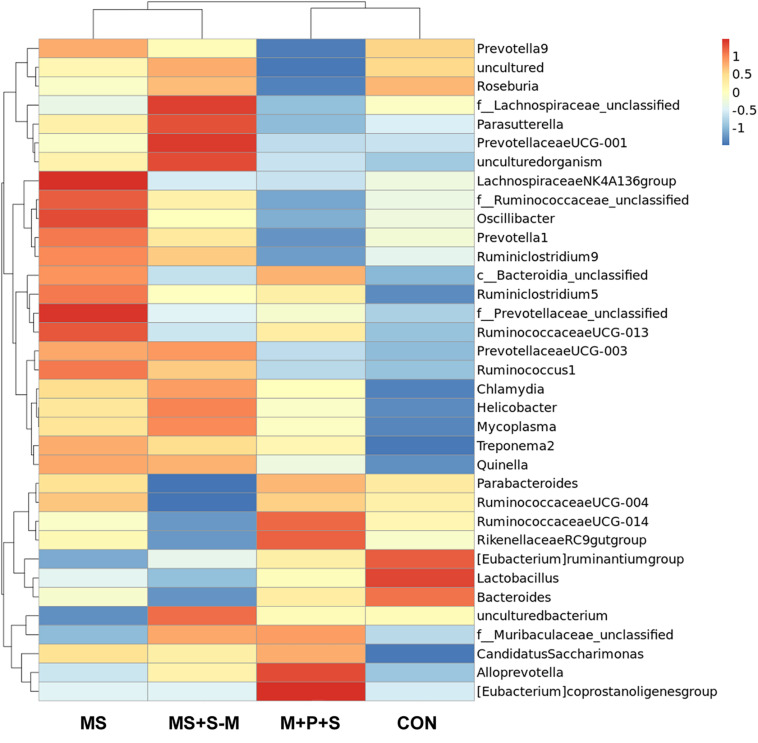
Cluster heatmap analysis of the top 35 most abundant fecal microbiota at the genus level in experimental groups, control (Con), maternally separated pups (MS), mother with pups and stranger (M+P+S), maternally separated pups and stranger (MS+S–M).

The heatmap analysis showed the relative abundance of differential bacteria at the genus level (Figure 2). The analysis classified the identified bacteria as two major clusters, cluster-1 further classified into two subclusters each with 5 and 7 genera, respectively. Similarly, cluster-2 was classified into two subclusters each with 16 and 7 genera, respectively. Interestingly, a relative proportion of beneficial bacteria such as *Parabacteroides, Rikenellaceae, Eubacterium ruminantium, Bacteroides*, and especially *Lactobacillus* were enriched in cluster 1 of the control and M + P + S groups, but their abundance was less in the MS and MS+S−M groups. In contrast, the Lachnospiraceae cluster-2, *Parasutterella, Prevotellaceae, Oscillibacter, Ruminiclostridium*, and *Bacteroidia* bacterial sp. were highly present in the stressed group (MS; MS + S−M), but this presence was considerably less in the control and M+P+S group.

### *Lactobacillus* Supplementation Prevents the Early Life SSE Induced Anxiety-Like Behavior

**FIGURE 3:**
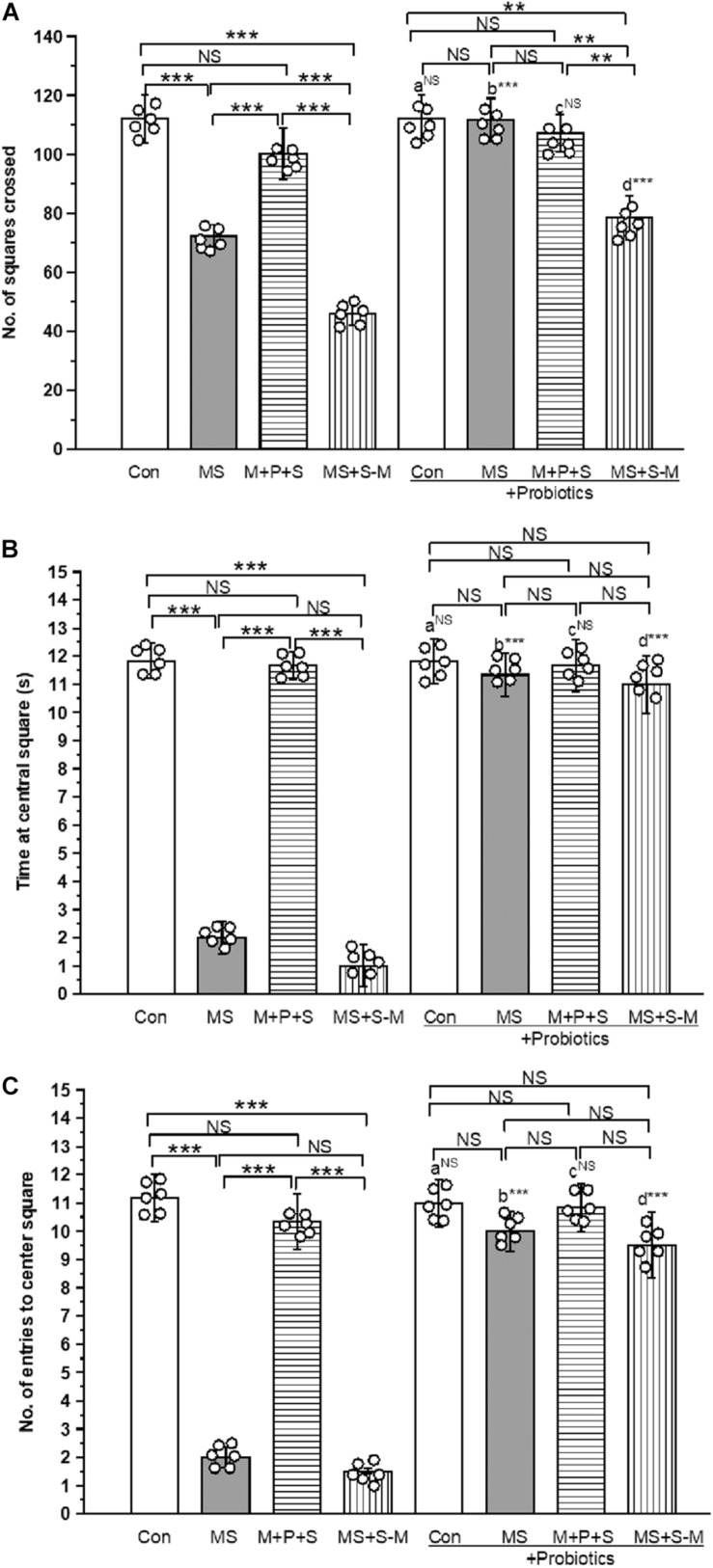
The open field test indicates that *Lactobacillus* supplementation prevented anxiety-like behavior induced by early life SSE. In the open-field test, the *Lactobacillus* supplemented group significantly crossed more squares **(A)**, spent more time at central square **(B)**, higher numbers of entries to the center square **(C)**. Values are presented as mean ± SEM. Significant differences between groups are indicated by ***P < 0.001, **P < 0.01; a: con vs. con + Pro; b: MS vs. MS + Pro; c: M + P + S vs. M + P + S + Pro; d: MS+S–M vs. MS+S–M + Pro; NS, not significant.

OFT-test was conducted to test anxiety-like behavior. We found a significant difference in the number of squares crossed by the individuals of the experimental group [*F*_(4,47)_ = 16.853, *P <* 0.001]. There was a significant interaction between the experimental groups × probiotic groups [*F*_(4,47)_ = 6.999, *P <* 0.001]. The control group significantly (*P <* 0.001) crossed more squares than the stressed groups (MS; M+P+S; MS+S−M). Among the stressed group, the M + P + S group significantly (*P <* 0.001) crossed more squares than the MS and MS+S−M groups. When we analyzed the number of squared crossed by the experimental rat pups, we found that there were no significant differences between the control and control + Pro groups (*P* = 0.066); similarly, the M + P + S and M+P+S+PRO groups were also not significantly different (*P* = 0.182). However, the MS + Pro (*P* < 0.001) and MS+S−M + Pro (*P* < 0.001) groups crossed more squares than the MS and MS+S−M groups, respectively (Figure 3A). Similarly, the time spent by individuals in the central square was significantly different between the experimental groups [*F*_(4,47)_ = 36.533, *P <* 0.001], and the interaction between the experimental groups x probiotic groups [*F*_(4,47)_ = 29.568, *P <* 0.001]. In comparison, the time spent by the control group in the center square was significantly longer (*P <* 0.001) than MS and MS+S−M. Among the stressed group, the M + P + S group spent significantly (*P <* 0.001) more time in the center square than the MS and MS+S−M groups. When we analyzed the time spent in the central square, we found that there were no significant differences between the control and control + Pro groups (*P* = 0.739) and, in parallel, there were no significant differences between the M + P + S and M+P+S + Pro groups (*P* = 0.335). However, the MS + Pro (*P* < 0.001) and MS+S−M + Pro (*P* < 0.001) group spent more time in the central square compared to the MS and MS+S−M groups, respectively (Figure 3B). Furthermore, the number of entries in the center square by the individuals was significantly different between the experimental groups [*F*_(4,47)_ = 19.560, *P <* 0.001]. There was a significant interaction between the experimental groups × probiotic groups [*F*_(4,47)_ = 6.906, *P <* 0.001]. The control group entered the center square significantly (*P <* 0.001) more than the MS and MS+S−M groups. Among the stressed group, the M + P + S group entered the center square significantly (*P <* 0.001) more than the MS and MS+S−M groups. When comparing the number of entries to the center square, there was no significant difference between the control group and the control + Pro group (*P* = 0.553); and between the M + P + S and M+P+S + Pro groups (*P* = 0.227). However, the MS + Pro (*P* < 0.001) and MS+S−M + Pro (*P* < 0.001) groups entered the center square significantly more often compared to the MS and MS+S−M groups, respectively (Figure 3C).

### *Lactobacillus* Supplementation Prevents Early Life SSE Induced Depressive-Like Behavior

**FIGURE 4:**
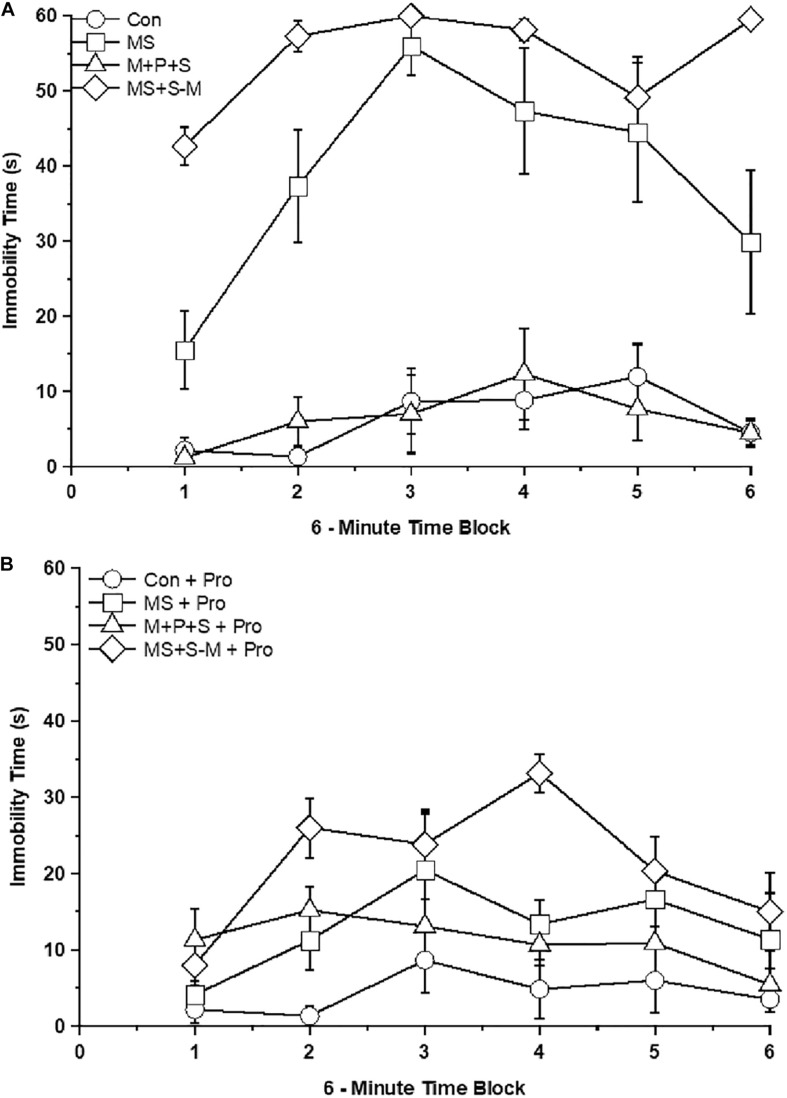
Tail suspension test in the stressed group or stressed group supplemented with probiotics. The duration of immobility was longer in the stressed groups compared to the control group and the stressed group supplemented with probiotics. Values are presented as mean ± SEM. **(A)** Early-life SSE induced immobilization and labels are indicated as ∘ – con; □ – MS; Δ - M+P+S; ⋄ - MS+S-M. **(B)** Supplementation of *Lactobacillus* significantly prevented the early-life SSE induced immobilization and labels are indicated as ∘ – con + Pro; □ – MS + Pro; Δ - M+P+S + Pro; ⋄ - MS+S-M + Pro.

Further analysis revealed a significant effect on immobility between the experimental groups [*F*_(4,47)_ = 54.197; *P* < 0.001] and interaction between the experimental groups × probiotic groups [*F*_(4,47)_ = 28.154; *P* < 0.001]. In comparison, MS (*P <* 0.001) and MS+S−M (*P <* 0.001) group individuals exhibited depression-like behavior with a longer immobility time than the control but not significantly different (*P* = 0.476) from the M + P + S group. Among the stressed group, the M + P + S group had significantly (*P <* 0.001) less immobile time than the MS and MS+S−M groups. There was a significant difference between the time period of 6 min in control and control + Pro groups (*P* < 0.001), MS and MS + Pro groups (*P* < 0.001), M+P+S and M+P+S + Pro groups (*P* < 0.001), MS+S−M and MS+S−M + Pro groups (*P* < 0.001). When we analyzed the immobility time between the control and the control + Pro group, there was no significant difference (*P* = 0.577) while there was a significant difference between the MS and MS + Pro groups (*P* < 0.001), M+P+S and M+P+S + Pro groups (*P* < 0.001), MS+S−M and MS+S−M + Pro groups (*P* < 0.001) (Figures 4A,B).

### *Lactobacillus* Supplementation Prevents the Early Life SSE Induced Changes in CORT and ACTH

**FIGURE 5:**
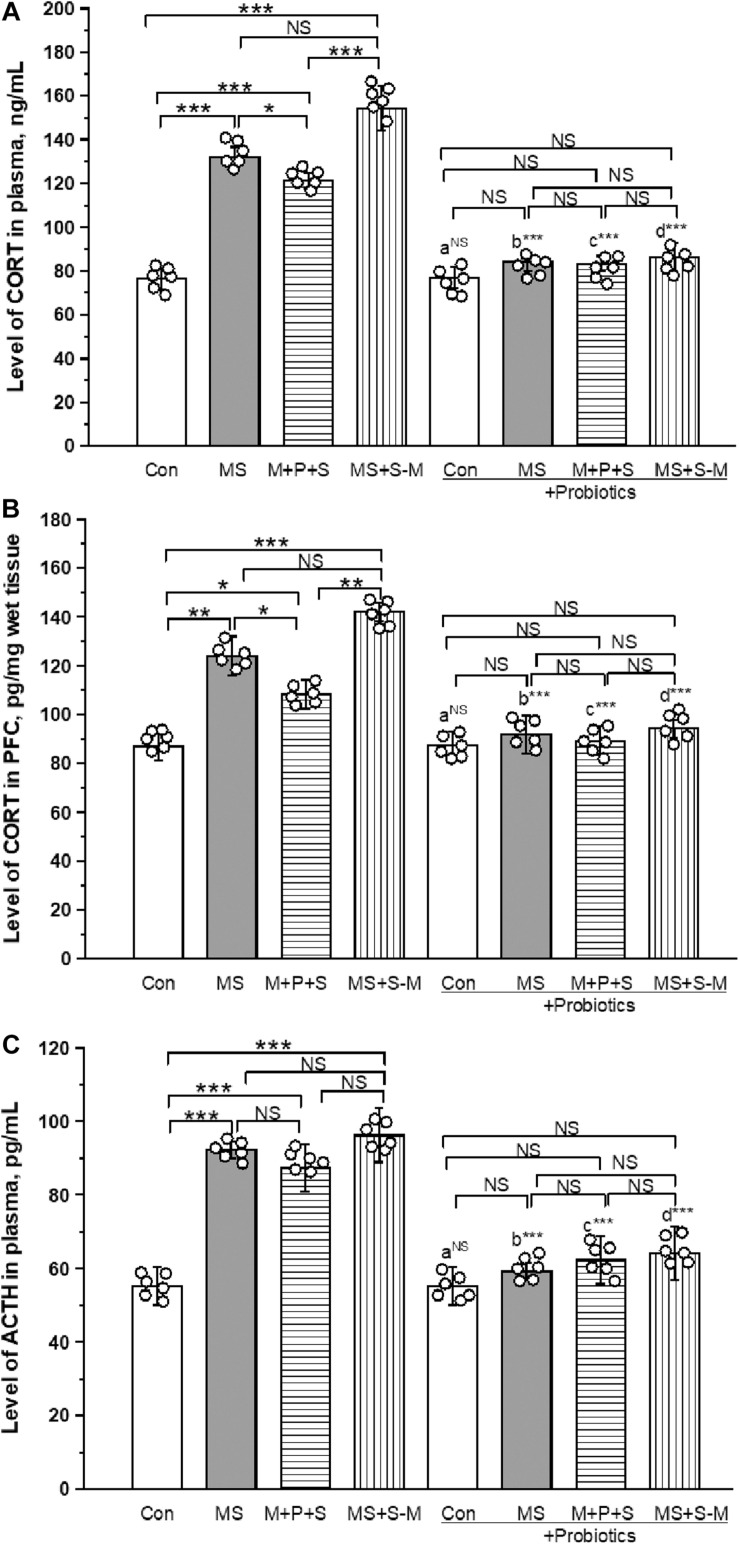
*Lactobacillus* supplementation significantly prevented early life SSE induced elevation of CORT in plasma **(A)**, PFC **(B)**, and plasma ACTH **(C)**. Values are presented as mean ± SEM. Significant differences between groups are indicated by ***P < 0.001, **P < 0.01, *P < 0.05; a: con vs. con + Pro; b: MS vs. MS + Pro; c: M + P + S vs. M + P + S + Pro; d: MS+S–M vs. MS+S–M + Pro; NS, not significant.

As shown in Figure 5A, a significant effect was observed in the level of CORT in plasma between the groups [*F*_(3,23)_ = 43.349; *P* < 0.001]. *The post hoc* test revealed that the level of CORT was significantly higher in MS (*P <* 0.001) and MS+S−M (*P <* 0.001) compared to the control. A higher level of CORT was detected in the MS (*P <* 0.05) and MS+S−M (*P <* 0.001) group compared to the M + P + S group. Compared to the control, the M + P + S group (*P <* 0.001) was significantly higher, while there were no significant differences between the MS and MS+S−M groups (*P* = 0.066). The estimated level of CORT in the probiotic-administrated groups was not significantly different between the experimental groups [*F*_(3,23)_ = 10.062; *P* = 0.124]. We also observed that the level of CORT in plasma did not make a significant difference between control and control + Pro groups (*P* = 0.841), while there was a significant difference between the MS and MS + Pro groups (*P* < 0.001), M+P+S, and M+P+S + Pro groups (*P* < 0.001), MS+S−M and MS+S−M + Pro groups (*P* < 0.001).

The estimated level of CORT in PFC was significantly different between the experimental groups [*F*_(3,23)_ = 18.521; *P* < 0.001]. *Post hoc* analysis detected a significantly higher level of CORT in the MS group (*P <* 0.001), M+P+S (*P <* 0.05) and MS+S−M (*P <* 0.001) group compared to the control. A significantly higher level was observed in the MS group (*P <* 0.05) and MS+S−M (*p <* 0.001) group than in the M + P + S group. There was no significant difference between the MS and MS+S−M groups (*P* = 0.081). *Lactobacillus* supplementation prevented early life SSE induced changes and there were no significant differences between the experimental groups [*F*_(3,23)_ = 1.750; *P* = 0.234]. Compared to the controls, we observed that the level of CORT in PFC did not make a significant difference between the Control and Control + Pro groups (*P* = 1.000). However, there were significant differences between the MS and MS + Pro groups (*P* < 0.001), M+P+S and M+P+S + Pro groups (*P* < 0.001), MS+S−M and MS+S−M + Pro groups (*P* < 0.001) (Figure 5B).

The level of ACTH in plasma between the experimental groups was significantly different [*F*_(3,23)_ = 10.843; *P* < 0.001]. *Post hoc* analysis suggests that the ACTH level was significantly higher in the MS (*P* < 0.001), M+P+S (*P* < 0.001), and MS+S−M (*P* < 0.001) groups compared to the control group. A significant difference was not detected for MS (*P* = 0.074), MS+S−M (*P* = 0.4000) compared to the M + P + S group. In the *Lactobacillus* supplemented groups, the estimated level was not significantly different between the experimental groups [*F*_(3,23)_ = 5.795; *P* = 0.092]. When we compared the level of ACTH in the control and control + Pro groups (*P* = 1.000), there was no significant difference (*P* = 0.349), while there was a significant difference between the MS and MS + Pro groups (*P* < 0.001), M+P+S and M+P+S + Pro groups (*P* < 0.001), MS+S−M and MS+S−M + Pro groups (*P* < 0.001) (Figure 5C).

### *Lactobacillus* Supplementation Prevents the Early Life SSE Induced Changes in Neurotransmitters

**FIGURE 6:**
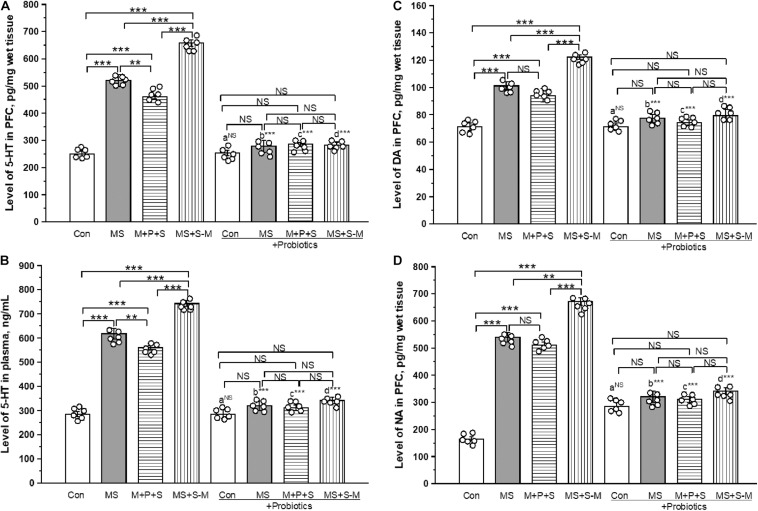
*Lactobacillus* supplementation prevented the effect of early life SSE on the level of 5-HT in PFC **(A)**, 5-HT in plasma **(B)**, DA in PFC **(C)**, and NA in PFC **(D)**. Values are presented as mean ± SEM. Significant differences between groups are indicated by ***P < 0.001, **P < 0.01, a: con vs. con + Pro; b: MS vs. MS + Pro; c: M + P + S vs. M + P + S + Pro; d: MS+S–M vs. MS+S–M + Pro; NS, not significant.

The estimated level of 5-HT in PFC was significantly different between the experimental groups [*F*_(3,23)_ = 26.846; *P* < 0.001] experiencing ELS. *Post hoc* analysis revealed that the 5-HT level in MS (*P* < 0.001), M+P+S (*P* < 0.001) and MS+S−M (*P* < 0.001) was significantly higher compared to the control. Further analysis revealed that the estimated level in the MS + S−M groups was significantly higher compared to the MS (*P* < 0.001) and M+P+S (*P* < 0.001) groups, while the estimated level was significantly higher in MS (*P* < 0.001) than in M + P + S. No significant differences were detected in the estimated 5-HT level in stressed groups supplemented with *Lactobacillus* [*F*_(3,23)_ = 1.794; *P* = 0.226]. The 5-HT level in PFC did not make a significant difference between the control and control + Pro groups (*P* = 1.000). There were significant differences between MS and MS + Pro groups (*P* < 0.001), M+P+S and M+P+S + Pro groups (*P* < 0.001), MS+S−M and MS+S−M + Pro groups (*P* < 0.001) (Figure 6A).

The estimated level of 5-HT in plasma was significantly different between the experimental groups [*F*_(3,23)_ = 12.481; *P* < 0.001]. Furthermore, *post hoc* analysis revealed that the 5-HT level was significantly higher in the MS group (*P* < 0.001), M+P+S (*P* < 0.001), and MS+S−M (*P* < 0.001) group than in the control. The estimated level in MS+S−M was significantly higher compared to MS (*P* < 0.001) and M+P+S (*P* < 0.001). There was no significant difference in 5-HT level between stressed groups supplemented with *Lactobacillus* [*F*_(3,23)_ = 3.208; *P* = 0.083]. Plasma 5-HT level did not show a significant difference between the control and control + Pro groups (*P* = 1.000). However, there were significant differences between MS and MS + Pro groups (*P* < 0.001), M+P+S and M+P+S + Pro groups (*P* < 0.001), MS+S−M and MS+S−M + Pro groups (*P* < 0.001) (Figure 6B).

The estimated level of DA in the PFC was significantly different between the groups [*F*_(3,23)_ = 14.004; *P* < 0.001]. *Post hoc* analysis described that the estimated level of DA in MS (*P* < 0.001), M+P+S (*P* < 0.001), and MS+S−M (*P* < 0.001) was significantly higher compared to control. The level of DA in MS (*P* < 0.05) and MS+S−M (*P* < 0.001) was significantly higher compared to the M + P + S group. The MS + S−M group (*P* < 0.001) reported a significantly higher level than when MS. *Lactobacillus* supplementation was prevented in early-life SSE induced changes, therefore, no significant differences were detected between the groups [*F*_(3,23)_ = 1.636; *P* = 0.257]. When comparing the level of DA in PFC, there were no significant differences between the control and control + Pro groups (*P* = 1.000). However, there were significant differences between the MS and MS + Pro groups (*P* < 0.001), M+P+S and M+P+S + Pro groups (*P* < 0.001), MS+S−M and MS+S−M + Pro groups (*P* < 0.001) (Figure 6C).

The level of NA was significantly different between the experimental groups [*F*_(3,23)_ = 6.959; *P* < 0.001]. *Post hoc* analysis showed that the estimated levels of NA in MS (*P* < 0.001), M+P+S (*P* < 0.001), and MS+S−M (*P* < 0.001) were significantly higher than in the control. Similarly, MS (*P* < 0.033) and MS+S−M (*P* < 0.001) was significantly higher compared to the M + P + S group. In the *Lactobacillus-supplied* groups, the analysis did not detect a significant difference between the experimental groups [*F*_(3,23)_ = 3.528; *P* = 0.068]. The level of NA in the PFC did not show significant differences between the control and control + Pro groups (*P* = 1.000). However, there were significant differences between the MS and MS + Pro groups (*P* < 0.001), M+P+S and M+P+S + Pro groups (*P* < 0.001), MS+S−M and MS+S−M + Pro groups (*P* < 0.001) (Figure 6D).

### *Lactobacillus* Supplementation Prevents the Early Life SSE Induced Changes in miR-124 and -132 Expression

**FIGURE 7:**
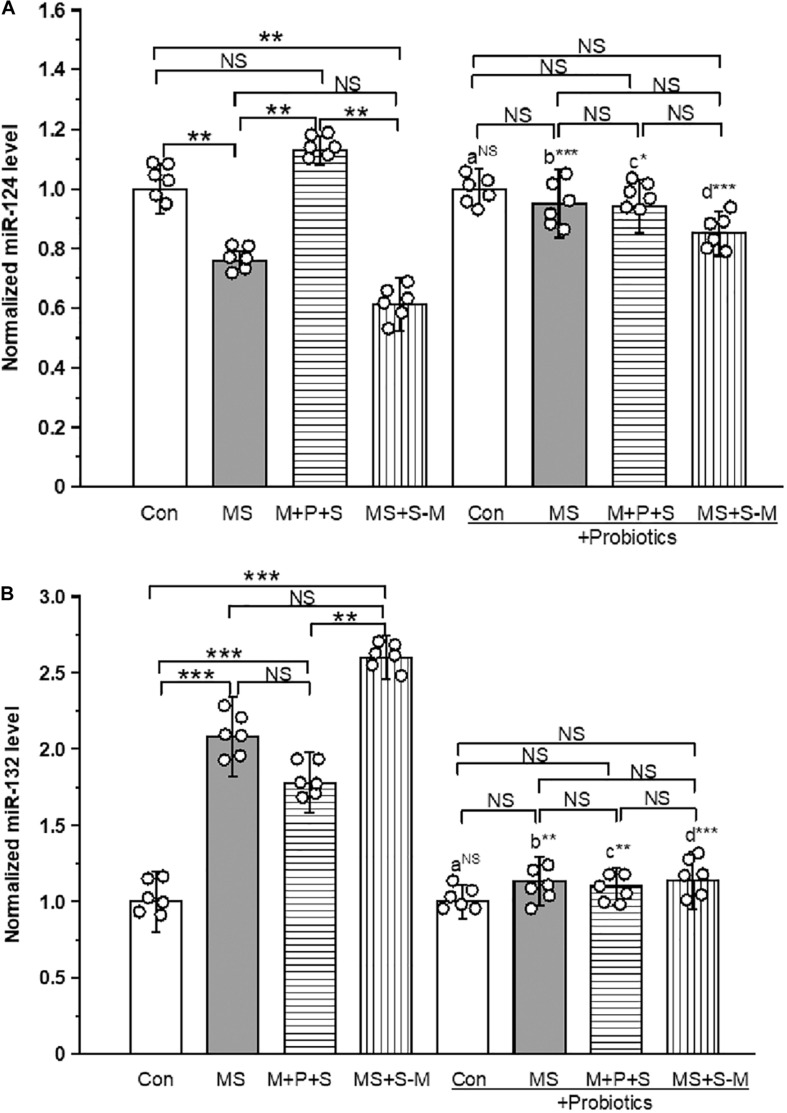
Early life SSE differently regulated the expression of miR-124 **(A)** and miR-132 **(B)**. Supplementation of *Lactobacillus* normalized the expression of miR-124 and miR-132 levels. Values are presented as mean ± SEM. Significant differences between groups are indicated by ***P < 0.001, **P < 0.01, *P < 0.05; a: con vs. con + Pro; b: MS vs. MS + Pro; c: M + P + S vs. M + P + S + Pro; d: MS+S–M vs. MS+S–M + Pro; NS, not significant.

The estimated level of miR-124 expression [*F*_(3,23)_ = 13.18; *P* < 0.001] expression was significantly different between the experimental groups. *Post hoc* analysis showed that the level of expression of miR-124 was significantly low in the MS (*P* < 0.005) and MS+S−M (*P* < 0.05) groups compared to the control and the M + P + S group. However, no significant differences were detected between the control and M+P+S (*P* = 1.000), and also between the MS and MS+S−M groups (*P* = 1.000). We did not detect a significant difference [*F*_(3,23)_ = 3.394; *P* = 0.074] between the experimental groups supplemented with *Lactobacillus.* When comparing the level of miR-124 expression, there were no significant difference between the control and control + Pro groups (*P* = 1.000). However, there were significant differences between the MS and MS + Pro groups (*P* < 0.001), M+P+S and M+P+S + Pro groups (*P* < 0.05), MS+S−M and MS+S−M + Pro groups (*P* < 0.001) (Figure 7A and Supplementary Figure 3).

Similarly, the level of expression of miR-132 was significantly different between the experimental groups [*F*_(3,23)_ = 16.964; *P* < 0.001]. *Post hoc* analysis revealed that the miR-132 level was significantly higher in the MS (*P* < 0.001), M+P+S (*P* < 0.001) and MS+S−M (*P* < 0.001) groups compared to the control. However, there were no significant differences between the MS (*P* = 1.000) and M+P+S (*P* = 1.000) and MS+S−M groups (*P* = 1.000). Further analysis showed that miR-132 expression levels in the experimental groups were not significantly different between the *Lactobacillus* supplemented groups [*F*_(3,23)_ = 3.678; *P* = 0.062]. The level of expression of miR-132 expression had no significant difference between the control and control + Pro groups (*P* = 0.441). However, there were significant differences between the MS and MS + Pro groups (*P* < 0.01), M+P+S and M+P+S + Pro groups (*P* < 0.01), MS+S−M and MS+S−M + Pro groups (*P* < 0.001) (Figure 7B and Supplementary Figure 3).

### *Lactobacillus* Supplementation Prevents Early Life SSE Induced Changes in the AMPA Receptor (GluR1 and GluR2), Glucocorticoid Receptors (GR) and Its Interaction

**FIGURE 8:**
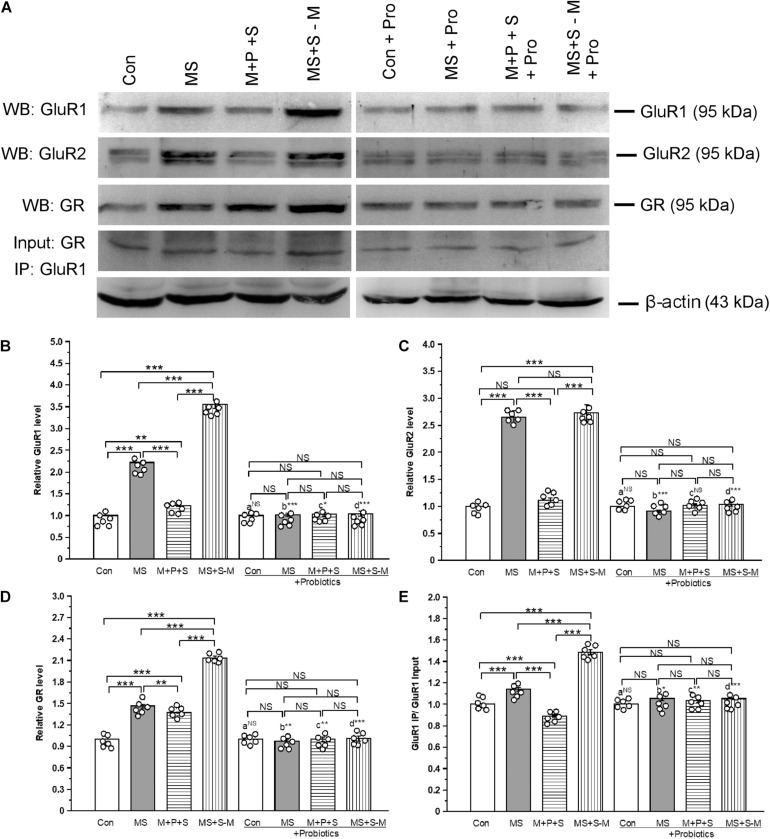
Early life SSE induced alteration in glutamate receptors (GluR1 and GluR2) and its interaction with glucocorticoid receptors (GR) prevented by lactobacillus supplementation. **(A)** Representative western blots showing immunoreactivity to GluR1, GluR2, GR, and GluR1 + GR Co-IP. Estimated level of GluR1 **(B)**, GluR2 **(C)**, GR **(D)**, and Co-IP of GluR1 with GR **(E)**. Values are presented as mean ± SEM. Significant differences between groups are indicated by ***P < 0.001, **P < 0.01, *P < 0.05; a: con vs. con + Pro; b: MS vs. MS + Pro; c: M + P + S vs. M + P + S + Pro; d: MS+S–M vs. MS+S–M + Pro; NS, not significant.

The estimated level of GluR1 showed that expression was significantly altered in the experimental groups [*F*_(3,23)_ = 43.2; *P* < 0.001] (Figure 8A). *Post hoc* analysis showed that the level of GluR1 was significantly higher in the MS (*P <* 0.001), M+P+S (*P <* 0.001), and MS+S−M (*P <* 0.001) groups compared to the control. However, the GluR1 level was significantly higher in MS+S−M compared to MS (*P <* 0.001) and M+P+S (*P <* 0.001). Furthermore, the analysis revealed that the GluR1 level was not significantly different between the experimental groups supplemented with *Lactobacillus* [*F*_(3,23)_ = 0.797; *P* = 0.529]. We observed that the level of GluR1 had no significant difference between the control and control + Pro groups (*P* = 1.000) while there were significant difference between the MS and MS + Pro groups (*P* < 0.001), M+P+S and M+P+S + Pro groups (*P* < 0.05), MS+S−M and MS+S−M + Pro groups (*P* < 0.001) (Figure 8B).

We found that the estimated level of GluR2 in the experimental groups was significantly different [*F*_(3,23)_ = 41.61; *P* < 0.001]. *Post hoc* analysis showed that the estimated level of GluR2 was significantly higher in the MS (*P* < 0.001) and MS+S−M (*P* < 0.001) groups compared to the control and M+P+S. There was no significant difference in GluR2 level between the control and M+P+S group (*P* = 0.203) and the MS and MS+S−M group (*P* = 1.000). Furthermore, the analysis revealed that the level of GluR2 was not significantly different between the experimental groups supplemented with *Lactobacillus* [*F*_(3,23)_ = 0.942; *P* = 0.465]. GluR2 level did not have significant difference between the control and control + Pro groups (*P* = 1.000) and, similarly, in the M + P + S and M+P+S + Pro groups (*P* = 0.072). Whereas, there were significant difference between MS and MS + Pro groups (*P* < 0.001), MS+S−M and MS+S−M + Pro groups (*P* < 0.001) (Figures 8A,C).

We found that the level of GR was significantly different between the experimental groups [*F*_(3,23)_ = 79.24; *P* < 0.001] (Figure 8A). *Post hoc* analysis revealed that the estimated level of GR was significantly higher in the MS (*P <* 0.001), M+P+S (*P <* 0.001), and MS+S−M (*P <* 0.001) groups compared to the control. However, the level of GR was significantly higher in MS+S−M than in the MS (*P <* 0.01) and M+P+S (*P <* 0.001) groups. The estimated level of GR was not significantly different between the *Lactobacillus* supplemented groups [*F*_(3,23)_ = 1.293; *P* = 0.342]. When we compared the level of GR, we found no significant difference between the control and control + Pro groups (*P* = 1.000). However, there were significant differences between the MS and MS + Pro groups (*P* < 0.01), M+P+S and M+P+S + Pro groups (*P* < 0.01), MS+S−M and MS+S−M + Pro groups (*P* < 0.001) (Figure 8D).

When we examined the interaction of GluR1 and GR, the analysis showed a significant difference in the interaction between the experimental groups [*F*_(3,23)_ = 22.84; *P* < 0.001] (Figure 8A). *Post hoc* analysis demonstrated that the detected level in the MS (*P <* 0.001) and MS+S−M (*P <* 0.001) group was significantly higher than the control and M+P+S. In comparison, the level was significantly higher in the MS + S−M group (*P <* 0.001) group than in the MS group. The analysis showed that there were no significant differences in the level of interaction of GluR1-GR in the *Lactobacillus* supplemented groups [*F*_(3,23)_ = 0.288; *P* = 0.833]. The level of GluR1-GR proteins had no significant difference between the control and control + Pro groups (*P* = 1.000). However, there were significant difference between the MS and MS + Pro groups (*P* < 0.05), M+P+S and M+P+S + Pro groups (*P* < 0.01), MS+S−M and MS+S−M + Pro groups (*P* < 0.001) (Figure 8E).

### *Lactobacillus* Supplementation Prevents Early Life SSE Induced Changes in the Expression of NMDA Receptors

**FIGURE 9:**
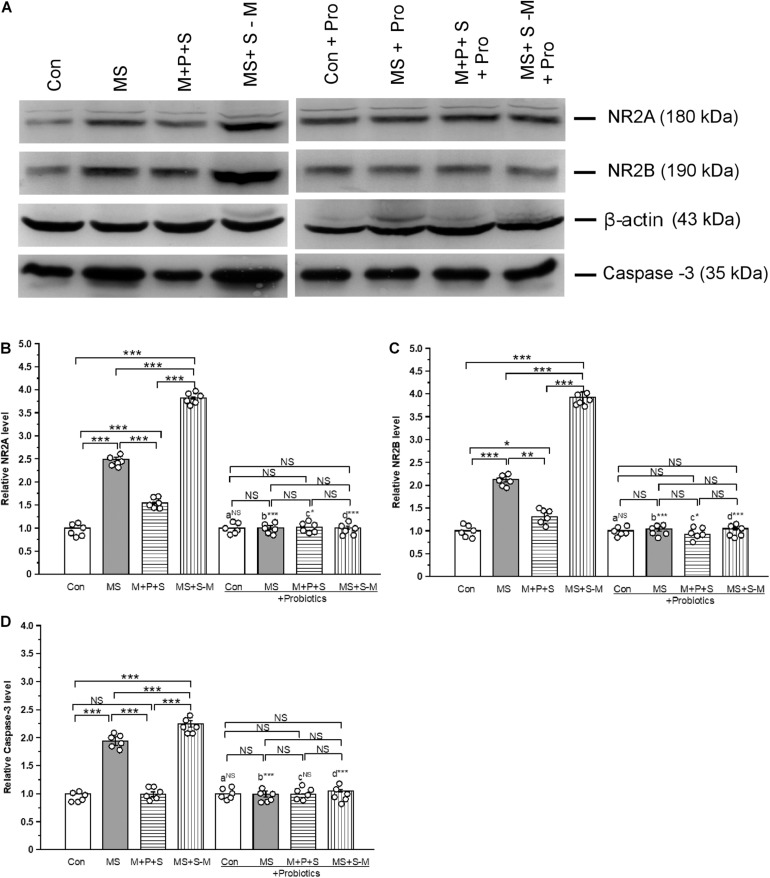
*Lactobacillus* supplementation prevented early-life SSE induced changes in NMDA receptors and caspase-3. Representative western blots showing the alternations in NR2A, NR2B, and Caspase-3 **(A)**. The relative level of expression of NR2A **(B)** and NR2B **(C)** and Caspase-3 **(D)** increased after the individuals experienced social stress. Values are presented as mean ± SEM. Significant differences between groups are indicated by ***P < 0.001, **P < 0.01, *P < 0.05; a: con vs. con + Pro; b: MS vs. MS + Pro; c: M + P + S vs. M + P + S + Pro; d: MS+S–M vs. MS+S–M + Pro; NS, not significant.

The estimated level of NR2A [*F*_(3,23)_ = 52.609; *P* < 0.001] was significantly different between the experimental groups (Figure 9A). *Post hoc* analysis suggests that the level of NR2A was significantly higher in the MS (*P* < 0.001), M+P+S (*P* < 0.001) and MS+S−M (*P* < 0.001) groups compared to the control. However, the estimated level was significantly higher in the MS + S−M group than in the MS group (*P* < 0.001) and M+P+S (*P* < 0.001) group. Compared to the M+P+S group, the NR2A level was significantly higher in MS (*P* < 0.001) than M+P+S group. Additionally, the estimated level of NR2A was not significantly different between the groups [*F*_(3,23)_ = 0.107; *P* = 0.953] in *the Lactobacillus-supplied* groups. The level of NR2A did not show significant difference between the control and control + Pro groups (*P* = 1.000). However, there were significant differences between the MS and MS + Pro groups (*P* < 0.001), M+P+S and M+P+S + Pro groups (*P* < 0.05), MS+S−M and MS+S−M + Pro groups (*P* < 0.001) (Figure 9B).

Similarly, the estimated level of NR2B was significantly different between the experimental groups [*F*_(3,23)_ = 73.43; *P* < 0.001]. *Post hoc* analysis detected that the level of NR2B was significantly higher in the MS (*P* < 0.001), M+P+S (*P* < 0.001), and MS+S−M (*P* < 0.001) groups compared to the control. But the level was significantly higher in MS+S−M than in MS (*P* < 0.001) and M+P+S (*P* < 0.001). The estimated level of NR2B in the *Lactobacillus* supplemented groups, the detected difference was not significantly different [*F*_(3,23)_ = 4.388; *P* = 0.082]. The level of NR2B did not have significant difference between the control and control + Pro groups (*P* = 1.000). However, there were significant difference between the MS and MS + Pro groups (*P* < 0.001), M+P+S and M+P+S + Pro groups (*P* < 0.05), MS+S−M and MS+S−M + Pro groups (*P* < 0.001) (Figures 9A,C).

### *Lactobacillus* Supplementation Prevents Early Life SSE Induced Changes in Caspase-3

Furthermore, the analysis revealed that the level of caspase-3 was significantly different between the experimental groups [*F*_(3,23)_ = 16.16; *P* < 0.001]. *Post hoc* analysis demonstrated that the caspase-3 level was significantly higher in the MS (*P* < 0.001) and MS+S−M (*P* < 0.001) groups compared to the control and M+P+S groups. However, the caspase-3 level was significantly higher in the MS + S−M (*P* < 0.001) than MS group, There was no significant difference between the control and the M+P+S group (*P* = 1.000). We found that the estimated level of Caspase-3 was not significantly different between the experimental groups supplemented with *Lactobacillus* [*F*_(3,23)_ = 0.873; *P* = 0.494]. The estimated level of Caspase-3 did not have significant difference between the control and control + Pro groups (*P* = 1000) and similarly, in the M + P + S and M+P+S + Pro groups (*P* = 0.977). However, there were significant differences between the MS and MS + Pro groups (*P* < 0.001), MS+S−M and MS+S−M + Pro groups (*P* < 0.001) (Figures 9A,D).

### *Lactobacillus* Supplementation Prevents Early Life SSE Induced Changes in Fecal Metabolites

**FIGURE 10:**
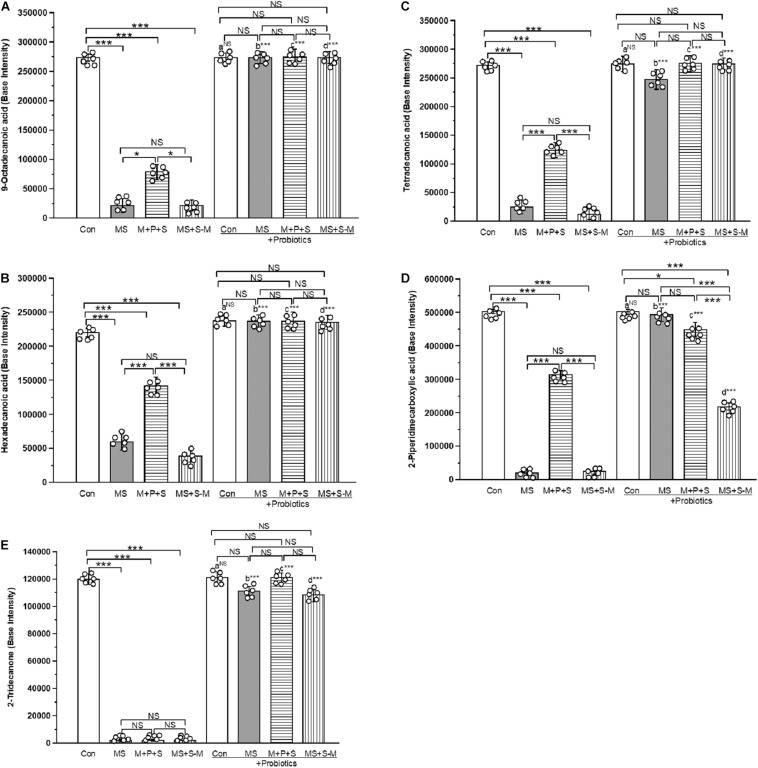
GCMS analysis revealed that early life SSE induced changes in metabolites. whereas, *Lactobacillus* supplementation prevented the effect on early life SSE in **(A)** 9-octadecanoic acid, **(B)** hexadecanoic acid, **(C)** tetradecanoic acid, **(D)** 2-piperidinecarboxylic acid, and **(E)** 2-tridecanone. Values are presented as mean ± SEM. Significant differences between groups are indicated by ***P < 0.001, *P < 0.05; a: con vs. con + Pro; b: MS vs. MS + Pro; c: M + P + S vs. M + P + S + Pro; d: MS+S–M vs. MS+S–M + Pro; NS, not significant.

A total of 438 metabolites were identified from fecal samples from the experimental groups. Of the identified compounds, 5 metabolites [2-Piperidine carboxylic acid (pipecolic acid), 9-Octadecanoic acid (oleic acid), hexadecanoic acid (palmitic acid), Tetradecanoic acid (myristic acid) and 2-Tridecanone (methyl undecyl ketone)] produced by *Lactobacillus* sp. were selected for the analysis. We performed selected ion monitoring (SIM) for the selected masses that are characteristic of the compound of interest in an expected retention time window to achieve both high sensitivity and high specificity. We compared the base intensity of the selected metabolic profile for all experimental groups (Figure 10 and Supplementary Figures 8–12). We found a similar profile pattern for the 9-octadecanoic acid (Figure 10A), hexadecanoic acid (Figure 10B), tetradecanoic acid (Figure 10C) and 2-piperidine carboxylic acid (Figure 10D) level was significantly low in MS (*P* < 0.001), M+P+S (*P* < 0.001) and MS+S−M group (*P* < 0.001) compared to the control group, and there was no significant difference between stressed groups. Whereas the basal level of these metabolites in the probiotic supplemented MS, M+P+S and MS+S−M groups was comparable to the control group and there was no significant difference between them. The level of 2-Tridecanone (Figure 10E) was significantly lower in the MS (*P* < 0.001), M+P+S (*P* < 0.001) and MS+S−M (*P* < 0.001) groups compared to the control, and there was no significant difference between the stressed groups (MS, M+P+S and MS+S−M) in any comparison. Probiotic supplementation elevated the level of 2-Tridecanone in the MS, M+P+S, and MS+S−M groups, which is similar to the control group. Therefore, no significant differences were detected between these groups. The base intensity of the metabolites (9-octadecanoic acid, hexadecanoic acid, tetradecanoic acid, 2-piperidine carboxylic acid and 2-tridecanone) did not have significant difference between the control and control + Pro groups (*P* = 1.000). However, there were significant difference between the MS and MS + Pro groups (*P* < 0.001), M+P+S and M+P+S + Pro groups (*P* < 0.001), MS+S−M and MS+S−M + Pro groups (*P* < 0.001).

## Discussion

The combination of the gut microbiota and diversity has been known to alter by several factors including social stress (O’Mahony et al., 2011; Fukui et al., 2018). In this study, we found that ELS during the hyporesponsive period induces dysbiosis, compared to the control group the estimated Fisher-alpha and chao-1 index diversity was lower in stressed groups. In support of this observation, previous studies have shown that host psychological stress shifts gut microbiota diversity, which is linked with the pathophysiology of anxiety/depressive disorder in animal models (Fukui et al., 2018; Partrick et al., 2018) and clinical reports (Naseribafrouei et al., 2014). Similar to earlier animal models and clinical reports (Clos-Garcia et al., 2019; Li et al., 2020; Xiang et al., 2020), our study showed that relative abundances of *Parasutterella, Oscillibacter, Ruminiclostridium, Treponema*, and *Helicobacter* were high in stressed groups compared to the control group. A parallel study (Ju et al., 2019) reported that the abundance of *Parasutterella* has been known to alter tryptophan and aromatic acid metabolism. In particular, colonization of *Oscillibacter* and *Clostridium* producing short chain fatty acids (Naseribafrouei et al., 2014; Yu et al., 2017), which is known to influence the expression of tryptophan hydroxylase (TPH), 5-HT synthesis, release and cause depression and anxiety (Spohn and Mawe, 2017). Similarly, the abundance of *Treponema* alters the serotonin transporter (El Aidy et al., 2017) and *Helicobacter* causes depression and affects cognitive function (Budzyński and Kłopocka, 2014). In contrast, the abundance of species such as *Lactobacillus, Bacteroides, E. ruminantium, Rikenellaceae*, and *Parabacteroides* was reduced in the stressed group compared to the control group. According to previous reports in animal models, the reduction in the abundance of *Lactobacillus, Bacteroides, E. ruminantium, Rikenellaceae*, and *Parabacteroides* was significantly associated with anxiety and depressive-like behavior (Bravo et al., 2011; Hsiao et al., 2013; Clos-Garcia et al., 2019). Considering the relationship between the relatively low and high abundance of these species with observed anxiety-like behavior, the results of our study are in line with those of other animal models (Budzyński and Kłopocka, 2014; El Aidy et al., 2017; Liang et al., 2017; Ju et al., 2019; Xiang et al., 2020) and clinical reports (Hashemi et al., 2016; Clos-Garcia et al., 2019; Li et al., 2020). However, probiotic supplementation has been known to cause host improvement (Bravo et al., 2011; Liang et al., 2015; Liao et al., 2019), and the *Lactobacillus* species is recognized as the most important probiotic in the intestinal microbiota, playing a key role in host health, including the attenuation of anxiety and cognitive improvement (Bercik et al., 2012). In this study, *Lactobacillus* supplementation resilience to anxiety-like behavior in open field test and depressive-like behavior in the tail suspension test was similar to other animal model studies (Bravo et al., 2011; Liang et al., 2015; Liao et al., 2019; McVey Neufeld et al., 2019). Stress-induced dysbiosis has been known to alter the homeostasis mechanism of stress hormones, neurotransmission, synaptogenesis, and associated behavior (Clarke et al., 2014). Overactivation of the HPA axis led to dysfunction of the feedback mechanism and altered the level of stress hormones (van Bodegom et al., 2017). In this study, we found that the basal level of CORT, ACTH, and expression of GR were elevated by ELS. An elevated level of stress hormone could be associated with observed anxiety-like/depressive behavior, as reported in other studies (Dinan and Cryan, 2012; Moloney et al., 2016; Fukui et al., 2018). When individuals in the stressed group were supplemented with *Lactobacillus*, the level of CORT, ACTH, and GR expression was maintained at the basal level. Whereas Lactobacillus supplementation protects HPA axis homeostasis (CORT, ACTH, and GR) in stressed individuals possibly through improving normal colonic development (Shi and Walker, 2004), gut barrier function (Zareie et al., 2006), and positively influencing the gut microbiota (Gareau et al., 2007; Bercik et al., 2012; Liang et al., 2015; McVey Neufeld et al., 2019).

Earlier studies have demonstrated that the cross-regulation of the HPA axis and neurotransmitters. 5-HT is considered a key neurotransmitter that participates in the brain-gut axis (Kilkens et al., 2003), along with NA and DA (Hamon and Blier, 2013). Similar to other ELS studies (Clarke et al., 2013; Rentesi et al., 2013), the level of 5-HT, NA, and DA was significantly elevated in individuals from the stressed group. In a previous study, probiotic supplementation normalized stress-induced modulation in the level of 5-HT, DA, and NA (Liang et al., 2015). Thus, it is possible that Lactobacillus supplementation in stressed individuals can reduce stress-induced changes in 5-HT, DA, and NA levels. The restoration of neurotransmitter levels by *Lactobacillus* supplementation may influence neurotransmission (Clarke et al., 2013; Distrutti et al., 2019), and then reduce anxiety and depressive-like behavior (Desbonnet et al., 2010; Liang et al., 2015).

In addition to the significant contribution of stress hormones (Moloney et al., 2016; Fukui et al., 2018), small non-coding RNAs (Clarke et al., 2013), especially microRNAs, could contribute to anxiety, depression, and anti-depression actions (Higuchi et al., 2016). Among many miRNAs, the participation of miR-124a/132 is strongly associated with stress-induced anxiety and depressive-like behavior (Higuchi et al., 2016; Aten et al., 2019). We found that ELS relatively suppresses miR-124a and up-regulates miR-132 expression like other animal models (Kawashima et al., 2010), which may interfere with GR expression (Vreugdenhil et al., 2009). Therefore, the individuals in the stressed group showed anxiety and depressive behavior. When stressed individuals were supplemented with *Lactobacillus*, the expression of miR-124a/132 was maintained at a normal level and comparable to that of the control. Supplementation of probiotics directly or through other regulatory mechanisms may control the expression of miRNAs (Demont et al., 2016). The observed behavior in the probiotic treated groups was made possible by the interaction of miRNAs with GR.

Increasing evidence also suggests that ELS produces long-lasting changes in the expression of AMPA and NMDA receptors (Ryan et al., 2009; Martisova et al., 2012) by an elevated level of CORT (Martin et al., 2009). We found that the expression of the receptor subunits of AMPA (GluR1 and GluR2) and NMDA (NR2A and NR2B) increased in stressed groups. Stress-induced elevation of CORT has been known to influence the release of glutamate/GABA through GR (Martisova et al., 2012). In this study, we found that there was interaction and that the level of interaction of GR-GluR1 was lower in the probiotic supplemented group (Martin et al., 2009; Martisova et al., 2012; Baj et al., 2019). The observed upregulation of NR2A, B and GluR1 receptors possibly occurs through activation of miR-132 during ELS (Kawashima et al., 2010), which may be associated with up-regulation of NMDA and AMPA receptors possibly through miR-132-MeCP2 regulation by ELS (Klein et al., 2007; Karen and Rajan, 2019). While probiotic supplementation normalized the expression of the NMDA and AMPA receptors in the stressed group (Baj et al., 2019), the balance of the gut microbiota could influence glutamate metabolism (Clos-Garcia et al., 2019). Furthermore, the over-activation of AMPA and NMDA receptors leads to neuronal death (Glazner and Mattson, 2000). Consequently, caspase-3 was activated in individuals in the stressed group, but *Lactobacillus* supplementation suppressed caspase-3 activation.

An earlier study has described how the low-molecular-weight metabolites produced by the gut microbiota are considered to be a marker of pathogenesis (De Angelis et al., 2013). The reduced level of metabolites (Hexadecanoic acid, 9-Octadecanoic acid, Tridecanoic acid, and Tetradecanoic acid) could be associated with the anxiety and depressive-like behavior observed in this study (Bernal-Morales et al., 2017; Kotlega et al., 2020). In agreement with earlier reports (Contreras et al., 2011, 2014), identified metabolites are restored to the basal level in individuals supplemented with *Lactobacillus* and showed anxiolytic behavior. The observed anxiolytic behavior in the *Lactobacillus* supplemented group could influence the HPA axis and the glucocorticoid stress response and also neurotransmission (Sarkar et al., 2016). Similarly, another metabolite 2-piperidine carboxylic acid was significantly low in the stressed group, while probiotic supplementation reverses the stress-induced effect. 2-piperidine carboxylic acid may act as a 5-HT reuptake inhibitor and lead to anxiolytic and antidepressant activity in individuals in the stressed group (Siddiqui et al., 2011), in contrast to its action on NMDA neurotransmission elicited depressive behavior (Sagratella et al., 1992). Furthermore, *Lactobacillus* supplementation in the stressed group restored the depleted metabolites (Stefanovic et al., 2017; Ming et al., 2018) to basel level, then ameliorated anxiety and depressive behavior.

## Conclusion

The present study suggests that the ‘hyporesponsive period’ in early life is a critical period that shapes the intestinal microbiota, thus early life SSE induces dysbiosis. Changes in the gut microbiota possibly alter the bidirectional communication of the microbiota-gut-brain axis through stress hormones, neurotransmitters, miRNAs, and glutamate receptors. Furthermore, changes in the abundance of the gut microbiota possibly imbalance the concentrations of neuroactive metabolites, which are linked to their physiological function and observed behavior. Interestingly, lactobacillus supplementation to the stressed group normalized the effect of SSE induced changes and associated behaviors. The results obtained from this study provide additional evidence to existing data and support our hypothesis that probiotic supplementation may be an additional strategy for the safe and effective treatment of anxiety-like behavior.

## Data Availability Statement

The datasets generated for this study can be found in online repositories. The names of the repository/repositories and accession number(s) can be found below: European Nucleotide Archive, PRJEB45530.

## Ethics Statement

The animal study was reviewed and approved by Institutional Animal Ethical Committee (IAEC), Bharathidasan University, Tiruchirappalli, India, following the guidelines of Committee for the Purpose of Control and Supervision of Experiments on Animals (CPCSEA), Government of India, India.

## Author Contributions

KR conceived and designed the experiments. CK performed the experiments. KR, CK, and DS analyzed the data. KR and DS contributed reagents, materials, and analysis tools. KR and CK drafted the manuscript. All authors contributed to the article and approved the submitted version.

## Conflict of Interest

The authors declare that the research was conducted in the absence of any commercial or financial relationships that could be construed as a potential conflict of interest.

## Publisher’s Note

All claims expressed in this article are solely those of the authors and do not necessarily represent those of their affiliated organizations, or those of the publisher, the editors and the reviewers. Any product that may be evaluated in this article, or claim that may be made by its manufacturer, is not guaranteed or endorsed by the publisher.
