# Supplementary material for: Lactobacillus paracasei Supplementation Prevents Early Life Stress-Induced Anxiety and Depressive-Like Behavior in Maternal Separation Model-Possible Involvement of Microbiota-Gut-Brain Axis in Differential Regulation of MicroRNA124a/132 and Glutamate Receptors
Source: Front Neurosci. 2021 Aug 31;15:719933. doi: 10.3389/fnins.2021.719933 (PMC8438336; doi:10.3389/fnins.2021.719933)
Supplement: Supplementary file 1 [file Data_Sheet_1.PDF]

Supplementary Informations

Lactobacillus paracasei Supplementation Prevents Early-Life Stress Induced Anxiety and Depressive-like Behaviour in Maternal Separation Model-Possible involvement of Microbiota-gut-Brain Axis in Differential Regulation of MicroRNA124a/132 and Glutamate Receptors

Christopher Karen[a], Douglas J. H. Shyu[b] and Koilmani Emmanuvel Rajan[a]*

[a]Behavioural Neuroscience Laboratory, Department of Animal Science, School of Life Sciences, Bharathidasan University, Tiruchirappalli 620024, India.

[b]Functional Genomics Laboratory, Department of Biological Science and Technology, National Pingtung University of Science and Technology, Neipu, Pingtung 912, Taiwan.

*Corresponding Author

Koilmani Emmanuvel Rajan

Behavioural Neuroscience Laboratory

Department of Animal Science,

Bharathidasan University, Tiruchirappalli -620024, India

Email: emmanuvel1972@yahoo.com

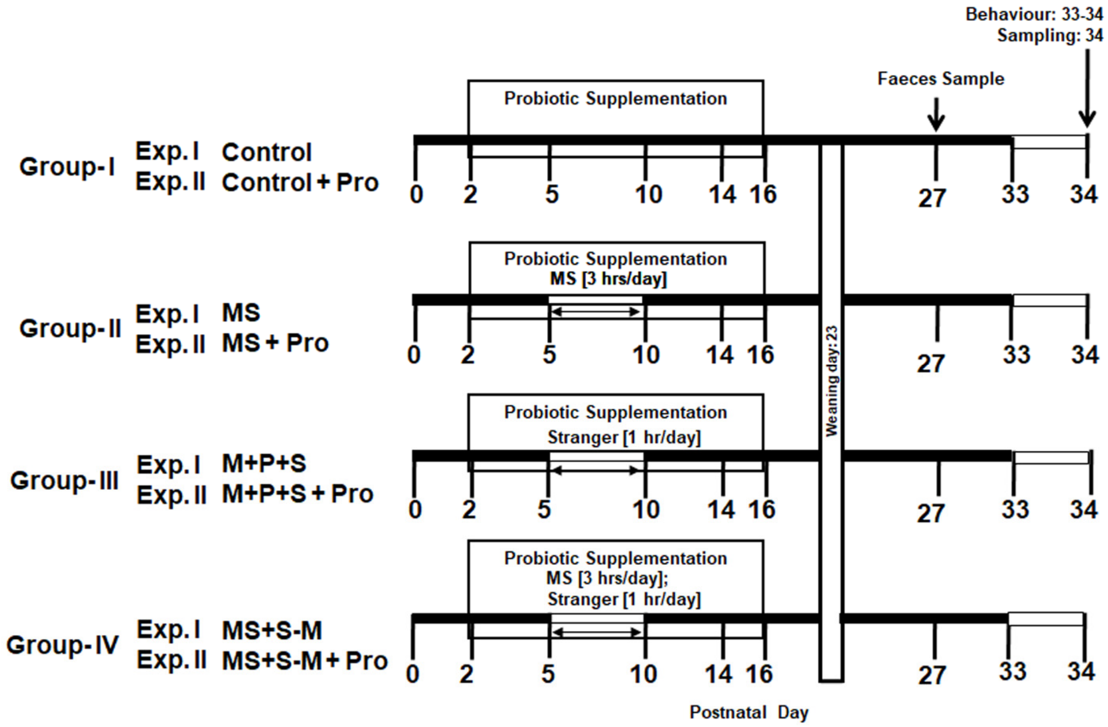

SUPPLEMENTARY FIGURE 1 Experimental timeline of events in the study, timeline showing the sequence of events and behavioural testing the animals underwent. Experiment initiated from postnatal day (PND) - 2 and terminated after behavioural testing on postnatal day (PND) - 34. Four groups of animals were used: (1) Control group pups and their mother (CON) were undisturbed upto PND-23, except during cleaning and general handling; (2) Maternal separation (MS) group: maternal separation was carried out for 3 hrs [09:00 - 12:00 hrs] from PND-5 to 10 by transfer the mother and their pups to another cage with home cage bedding and then mother was transferred back to the home cage immediately. Stressful social experience was provided by introducing the stranger (S) into their cage in the presence of mother with pups (M+P+S); or absence of mother (MS+S-M). Specially designed cage (standard laboratory cage: 43cm X 27cm X 15cm) – divided by wire mesh into two chambers, to prevent the physical contact of stranger (Senescence male; 18 months old) with the mother/ pups. The home cage

bedding was used during exposure to stranger in order to avoid additional stress and modification of maternal behaviour. One half of the specially designed cage with home cage bedding was used to place the experimental animals and the stranger was placed (10:00 – 11:00 hrs) in another half of the cage during PND-5 to 10.

METAGENOMICS 基因米克斯 Genomics | Home | Methods | Results | Genomics | Online Shop

Alpha Diversity

After careful filtering procedures, we were able to recapture a realistic richness of the microbial community, and identify meaningful differences in alpha and beta diversity in the samples. Microbial diversity can be evaluated within a community (**alpha diversity**: number of taxa within a single microbial ecosystem) or between the collection of samples (**beta diversity**: difference in taxonomic abundance profiles from different samples). The diversity of OTUs within a given sample is the alpha diversity of that sample. The simplest way of measuring it is to enumerate OTUs present in that sample, also called species richness. Here we provide related alpha diversity viewpoints including alpha diversity indices, rarefaction curves, rank abundance and venn diagram to evaluate the richness and diversity in the samples.

Alpha Indices Table

- Species Richness Estimator
  - Chao1
    - Estimate diversity from abundance data (importance of rare OTUs).
  - Observed species
    - Count of unique OTUs in each sample.
  - Good's coverage
    - Coverage = 1 - (number of individuals in species / total number of individuals)
    - For example, 0.96 means '4% of your reads in that sample are from OTUs that appear only once in that sample'.
  - Fisher's alpha
    - The relationship between the number of species and the number of individuals in those species.
- Species Evenness Estimator
  - Shannon
    - Shannon characterizes species diversity and accounts for abundance and evenness of the species.
  - Simpson
    - To describe the probability that a second individual drawn from a population should be of the same species as the first.
  - enspie
    - Effective number of species, probability of interspecific encounter (enspie is equivalent to '1 / dominance').

	chao1	enspie	shannon	simpson_reciprocal	fisher_alpha	goods_coverage	observed_species
C2	421	8.781200	4.923166	8.781200	64.30772	1	421
MS2	388	7.410636	4.501620	7.410636	58.43279	1	388
MSP_ST2	390	9.810218	4.946323	9.810218	56.88222	1	390
M_P_ST2	464	8.749482	5.102501	8.749482	70.54749	1	464

Note:
Observed Species estimates the amount of distinct OTUs found in each sample.

	chao1	enspie	shannon	simpson_reciprocal	fisher_alpha	goods_coverage	observed_species
C1	502	11.817373	5.472016	11.817373	77.09294	1	502
MS1	393	8.101643	4.673396	8.101643	60.12901	1	393
MSP_ST1	416	11.221958	5.174065	11.221958	59.93141	1	416
M_P_ST1	463	9.994057	5.334985	9.994057	72.13816	1	463

Note:
Observed Species estimates the amount of distinct OTUs found in each sample.

	chao1	enspie	shannon	simpson_reciprocal	fisher_alpha	goods_coverage	observed_species
C3	451	9.307646	5.055902	9.307646	66.85153	1	451
MS3	416	9.885369	5.156177	9.885369	62.24409	1	416
MSP_ST3	380	10.182126	4.891177	10.182126	56.19042	1	380
M_P_ST3	471	9.943845	5.237072	9.943845	71.19512	1	471

Note:
Observed Species estimates the amount of distinct OTUs found in each sample.

	chao1	enspie	shannon	simpson_reciprocal	fisher_alpha	goods_coverage	observed_species
C5	506	10.733751	5.233690	10.733751	80.04030	1	506
MS5	358	11.002581	4.971660	11.002581	53.29832	1	358
MSP_ST5	390	8.341680	4.847139	8.341680	58.34450	1	390
M_P_ST5	407	5.207505	4.091333	5.207505	59.62080	1	407

Note:
Observed Species estimates the amount of distinct OTUs found in each sample.

SUPPLEMENTARY FIGURE 2 Supporting evidence for manuscript Figure 1, showing the details of fecal microbiota. Details were used to calculate Chao-1 and Fisher –alpha Index.

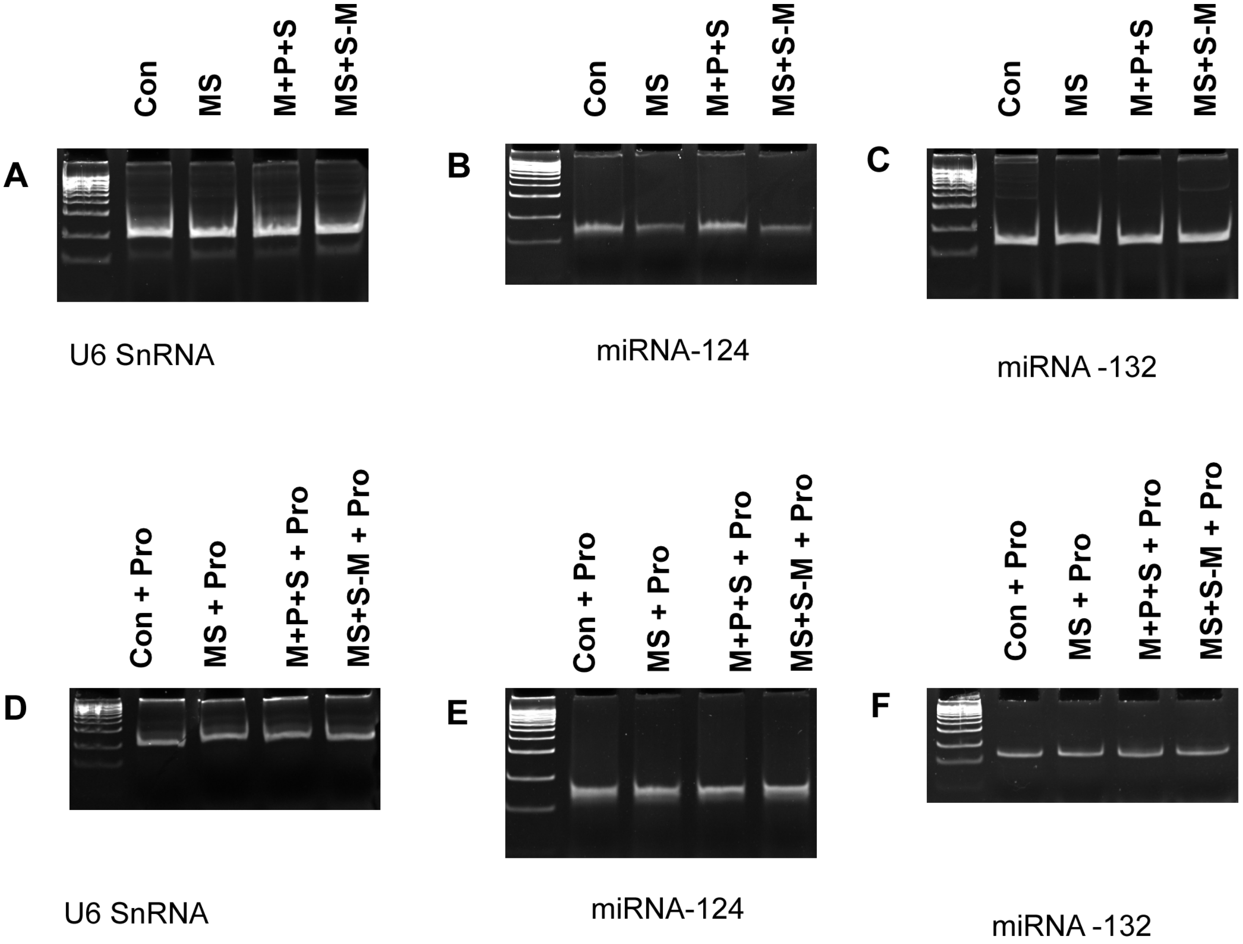

SUPPLEMENTARY FIGURE 3 Ethidium bromide stained native polyacrylamide gel (12%) showing the mRNA level of U6 SnRNA (94bp), miRNA-124 (56 bp), miRNA-132 (62bp) amplified from experimental groups and DNA marker (100 bp; Cat # 50020-1; Lucigen, Wisconsin, USA). Real-time PCR product is supporting for Figure 7 in the manuscript.

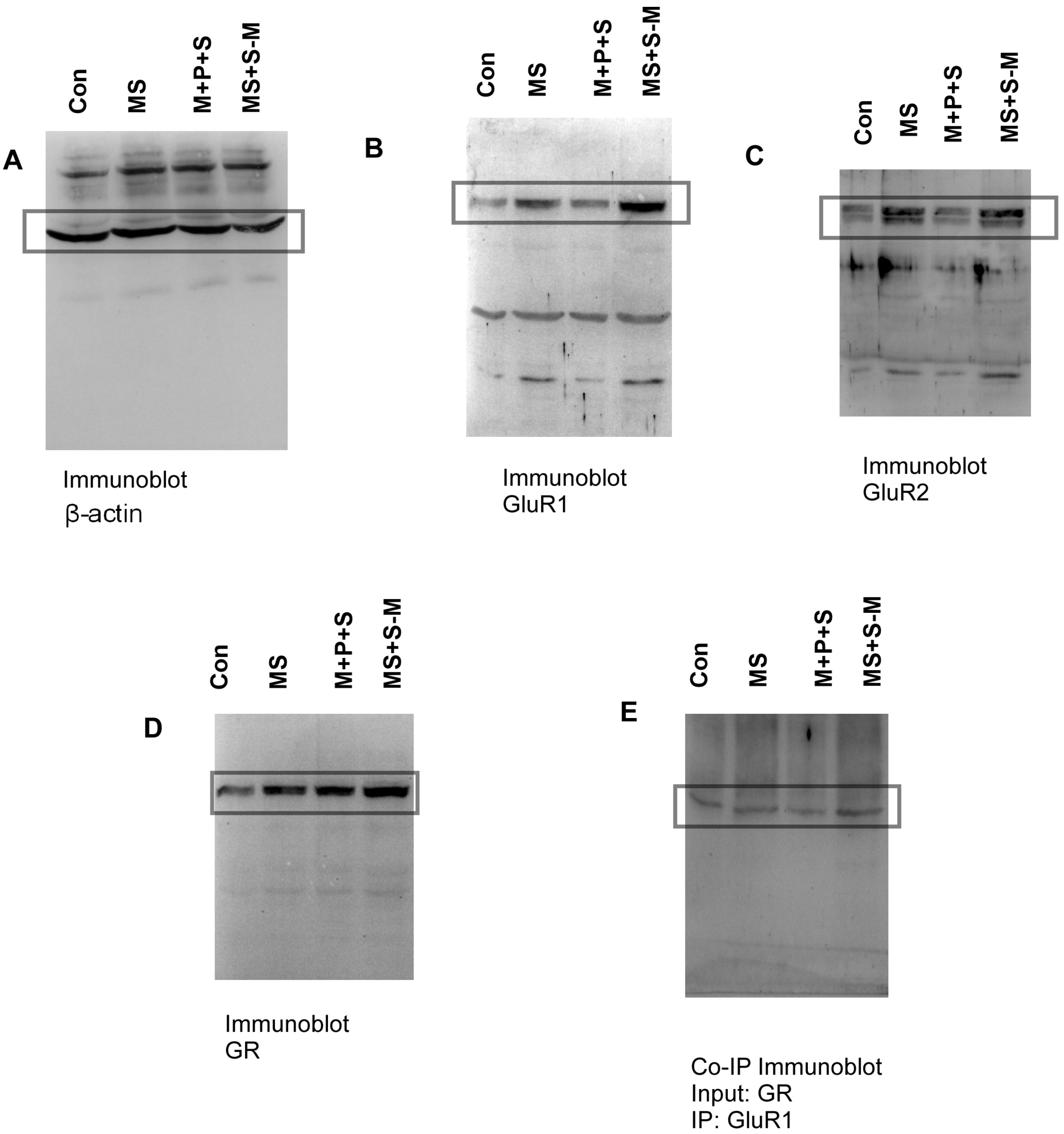

SUPPLEMENTARY FIGURE 4 Full immunoblot used for Figure 8 in the manuscript. Gray rectangles are the images cropped from each blot that are shown in the manuscript and each lane representing experimental groups (Con: Control; MS: Maternal separated pups; M+P+S: Mother and pups with stranger; MS+S-M: Maternal separated pups with stranger).

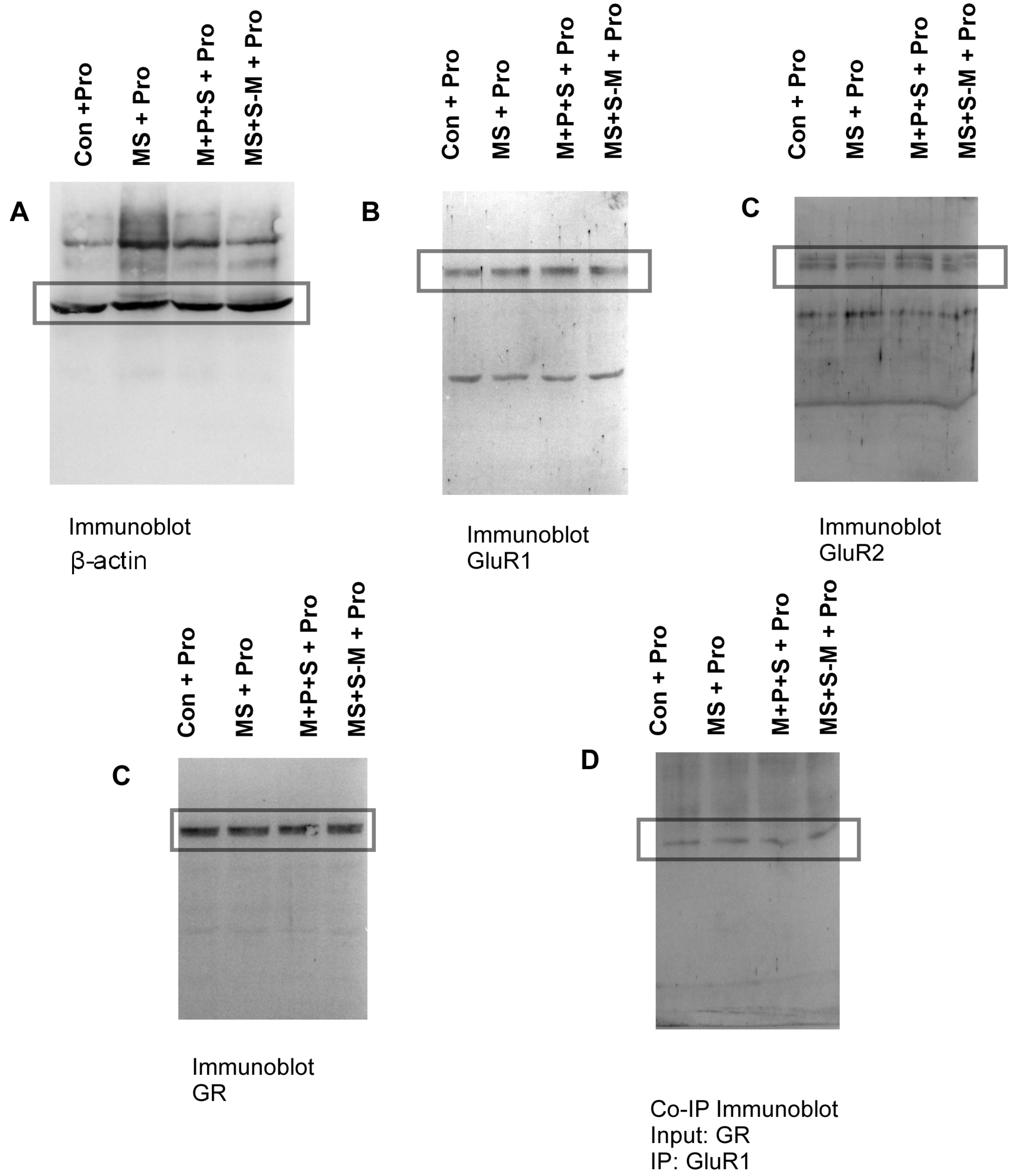

SUPPLEMENTARY FIGURE 5 Full immunoblots used for Figure 8 in the manuscript. Gray rectangles are the images cropped from each blot that are shown in the manuscript and each lane representing experimental groups supplemented with probiotics (Pro) [Con + Pro: Control ; MS + Pro: Maternal separated pups; M+P+S + Pro: Mother and pups with stranger; MS+S-M + Pro: Maternal separated pups with stranger].

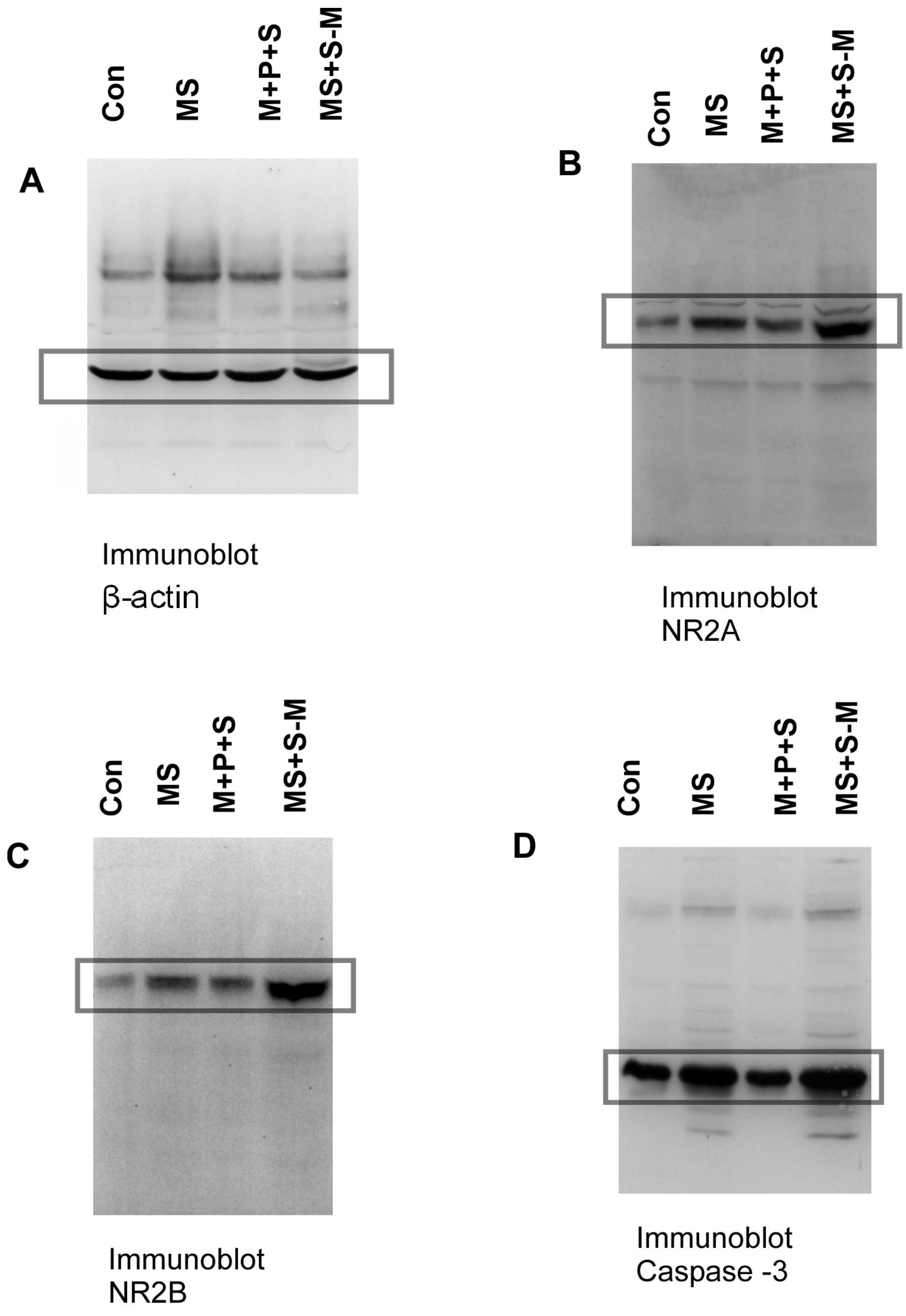

SUPPLEMENTARY FIGURE 6 Full immunoblot (uncropped) used for Figure 9 in the manuscript. Gray rectangles are the images cropped from each blot that are shown in the manuscript and each lane representing experimental groups (Con: Control; MS: Maternal separated pups; M+P+S: Mother and pups with stranger; MS+S-M: Maternal separated pups with stranger).

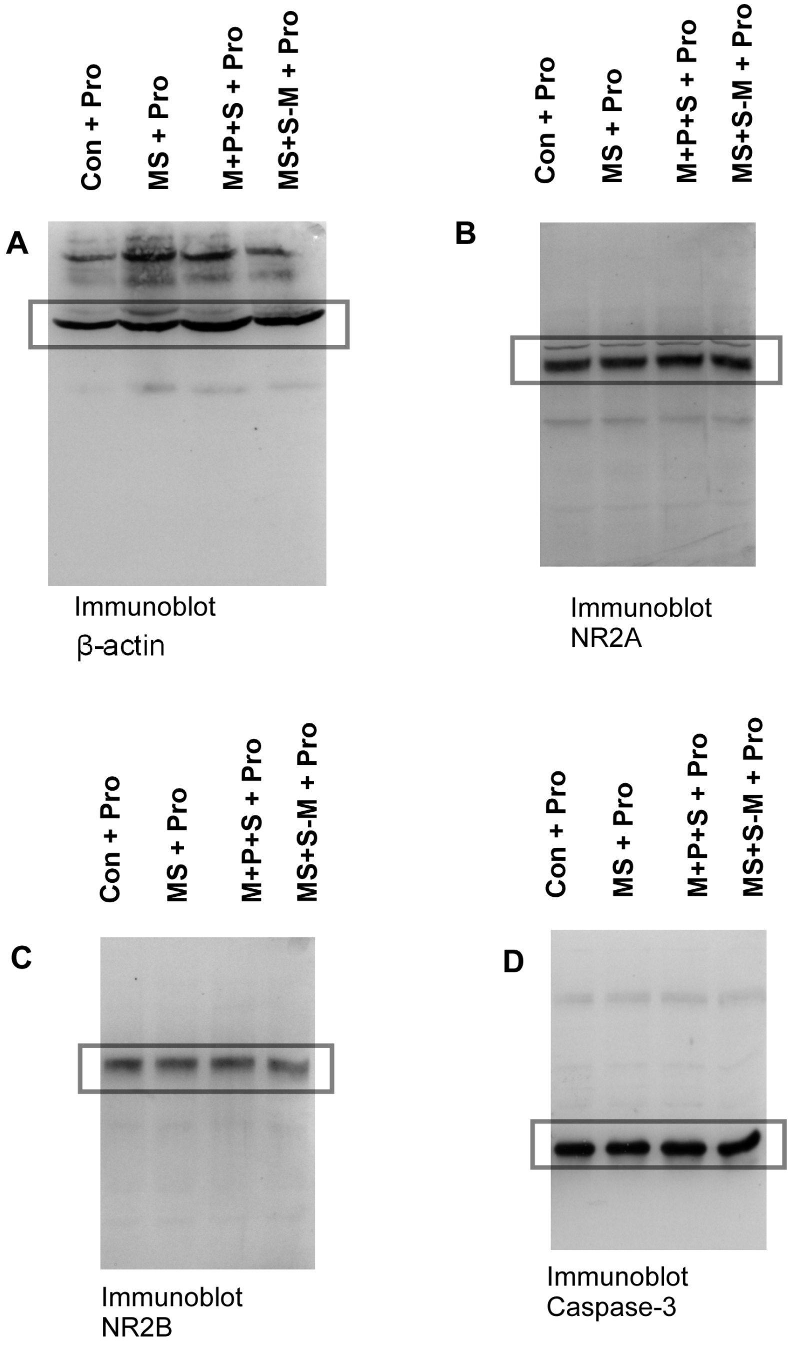

SUPPLEMENTARY FIGURE 7 Full immunoblot (uncropped) used for Figure 9 in the manuscript. Gray rectangles are the images cropped from each blot that are shown in the manuscript and each lane representing experimental groups supplemented with probiotics (Pro) [Con + Pro: Control ; MS + Pro: Maternal separated pups; M+P+S + Pro: Mother and pups with stranger; MS+S-M + Pro: Maternal separated pups with stranger].

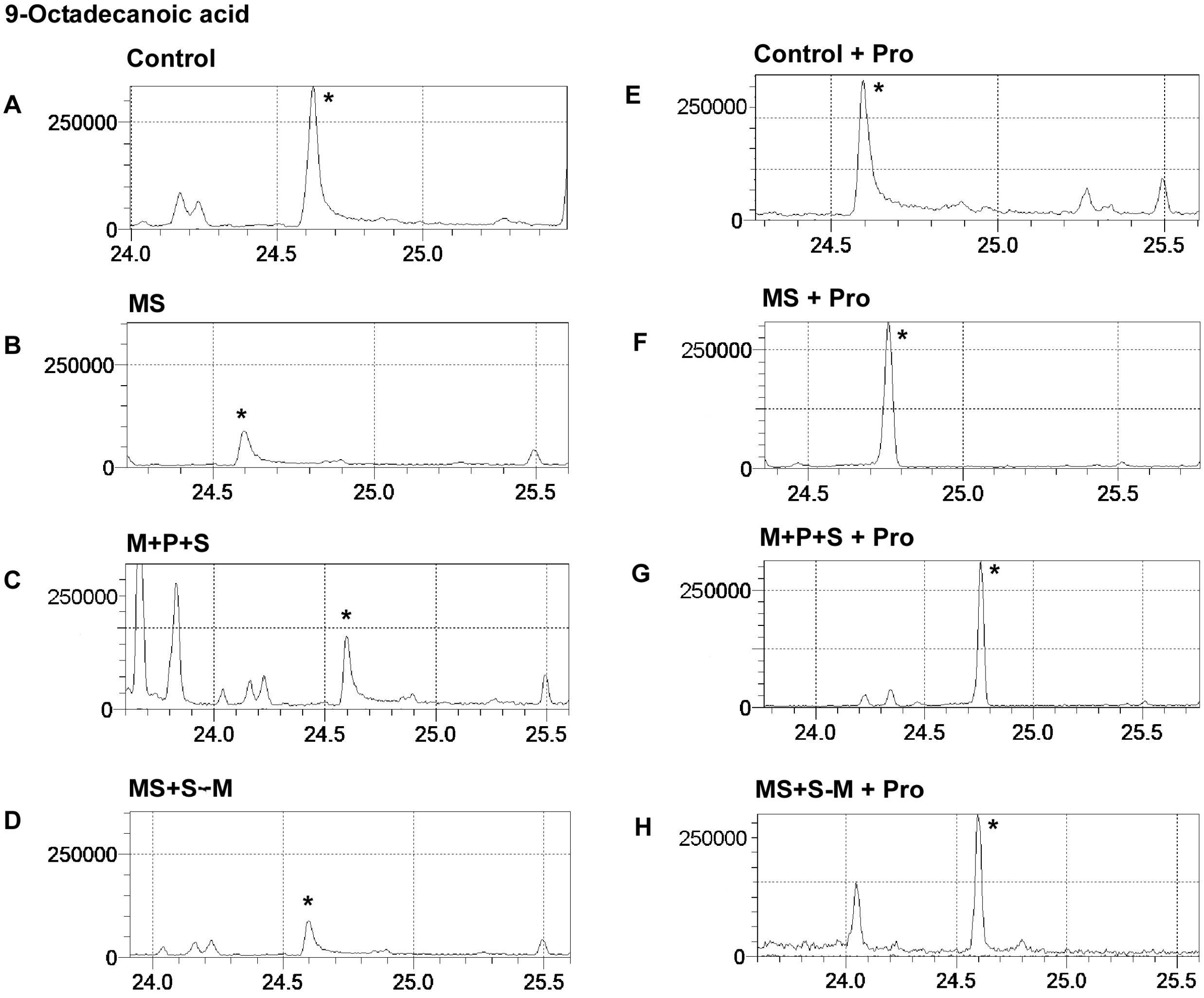

SUPPLEMENTARY FIGURE 8 GC-MS data for manuscript Figure 10A. Mass selective detector was run in scan mode to establish a fragmentation pattern of chromatogram and then using lab solutions GCMS postrun analysis software to get a specific peak (SIM chromatogram) of 9-Octadecanoic acid (retention time – 24.75 min, marked with *) with x-axis (retention time) and y-axis (absolute intensity). Experimental groups (A,B,C and D) and experimental group individuals are treated with *Lactobacillus*

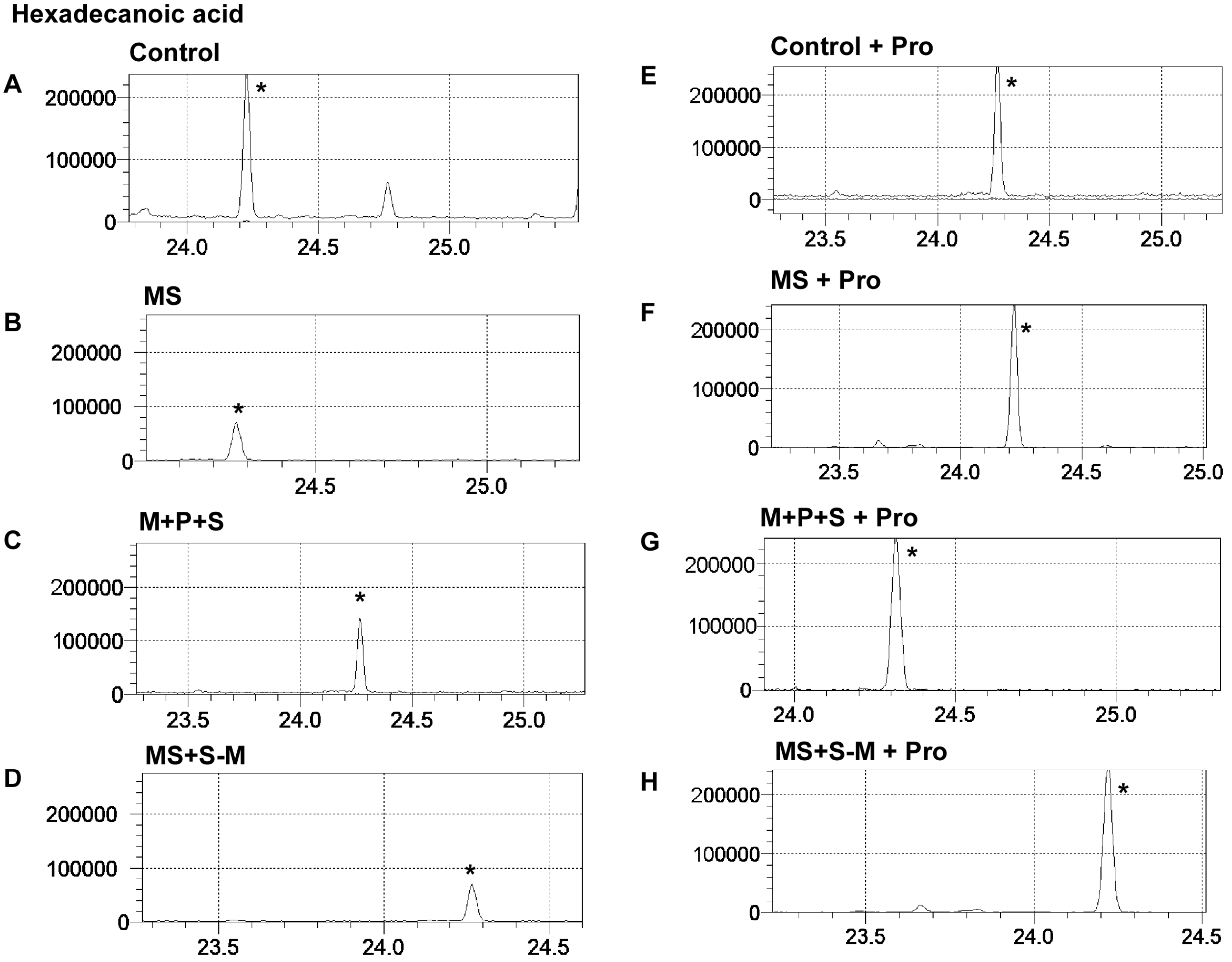

SUPPLEMENTARY FIGURE 9 GC-MS data for manuscript Figure 10B. Mass selective detector was run in scan mode to establish a fragmentation pattern of chromatogram and then using lab solutions GCMS postrun analysis software to get a specific peak (SIM chromatogram) of Hexadecanoic acid (retention time – 24.25 min; marked with *) with x-axis (retention time) and y-axis (absolute intensity). Experimental groups (A,B,C and D) and experimental group individuals are treated with *Lactobacillus* (E,F,G and H).

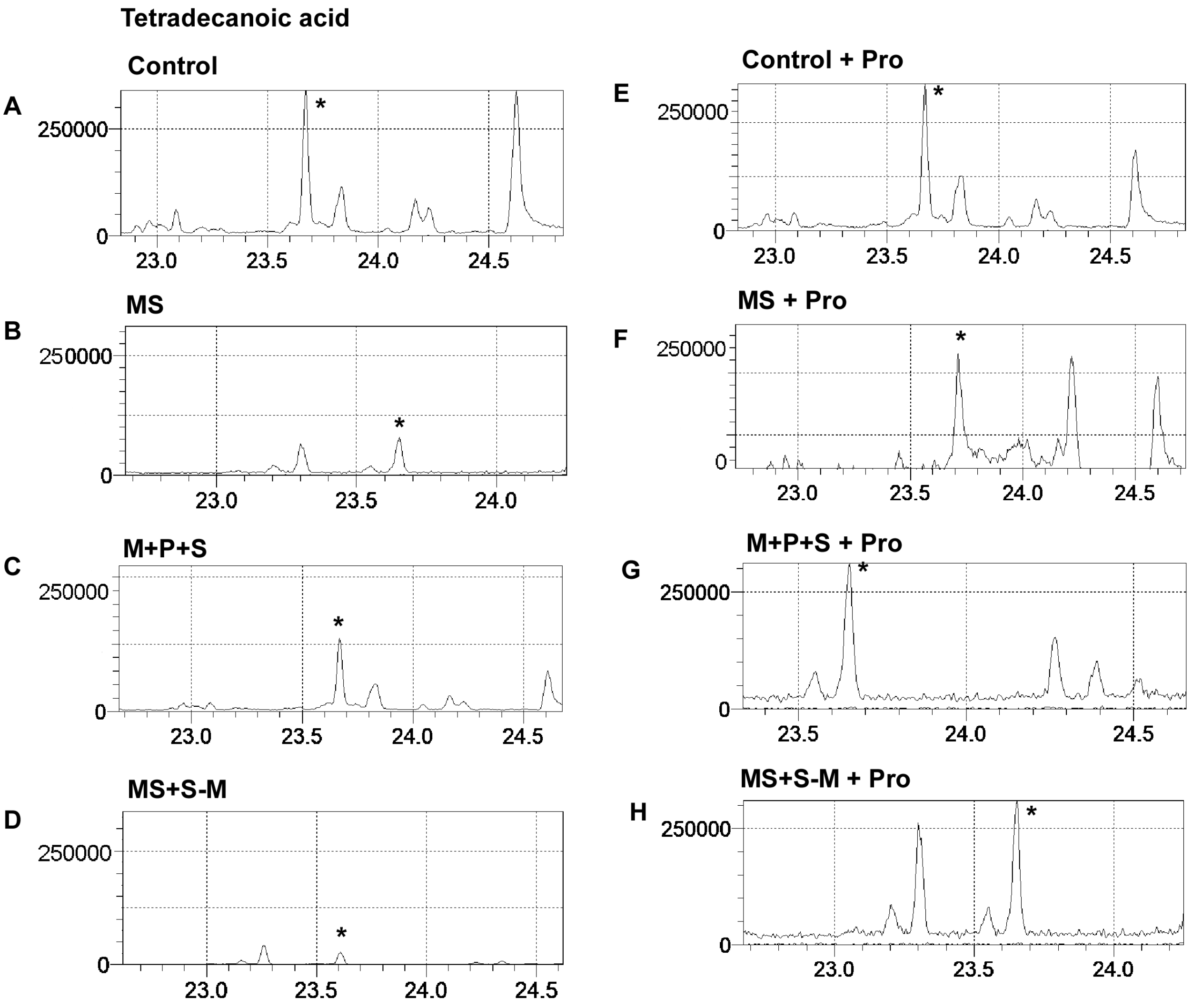

SUPPLEMENTARY FIGURE 10 GC-MS data for manuscript Figure 10C. Mass selective detector was run in scan mode to establish a fragmentation pattern of chromatogram and then using lab solutions GCMS postrun analysis software to get a specific peak (SIM chromatogram) of Tetradecanoic acid (retention time – 23.6 min; marked with *) with x-axis (retention time) and y-axis (absolute intensity). Experimental groups (A,B,C and D) and experimental group individuals are treated with *Lactobacillus* (E,F,G and H).

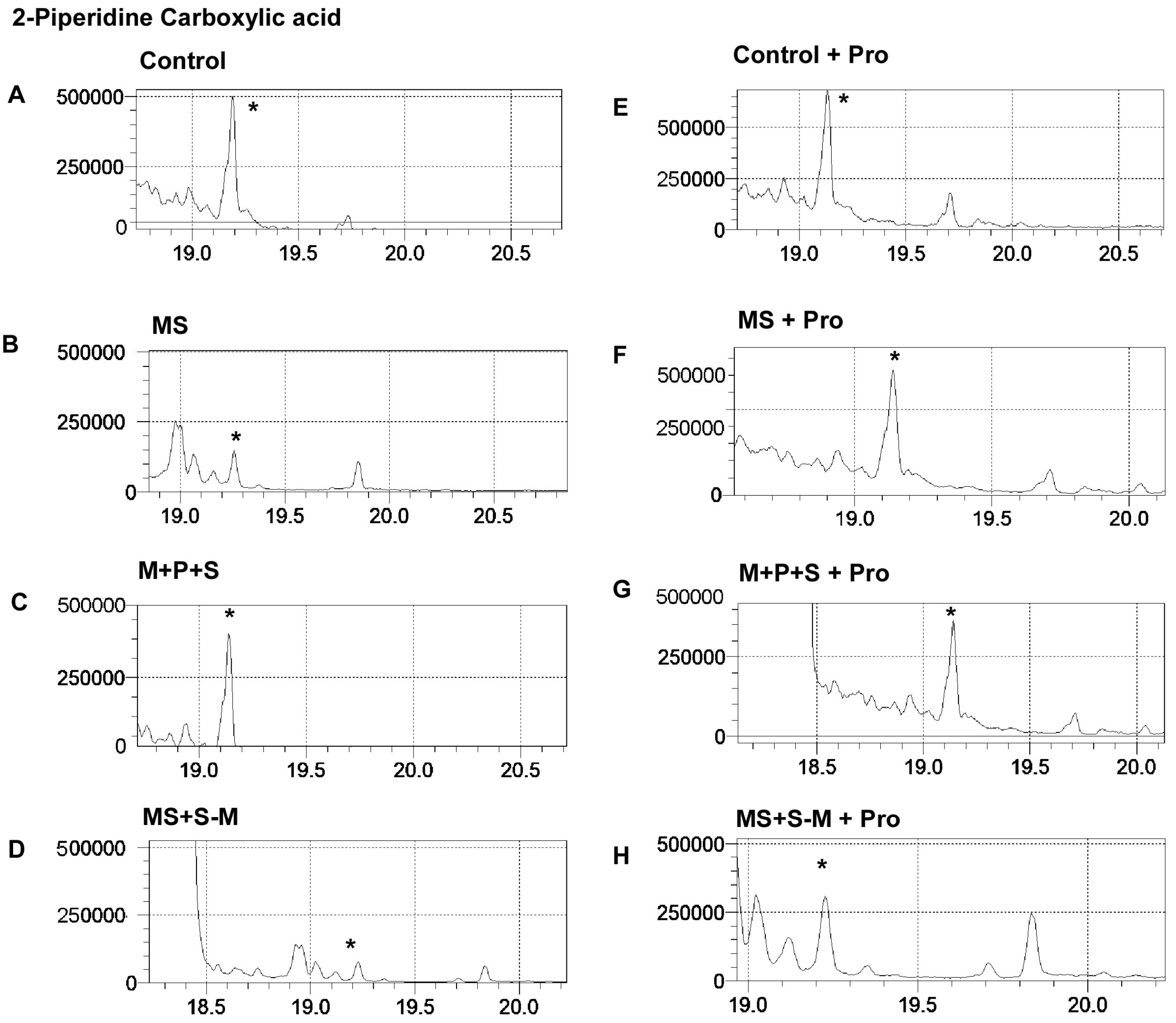

SUPPLEMENTARY FIGURE 11 GC-MS data for manuscript Figure 10D. Mass selective detector was run in scan mode to establish a fragmentation pattern of chromatogram and then using lab solutions GCMS postrun analysis software to get a specific peak (SIM chromatogram) of 2-Piperidinecarboxylic acid (retention time – 19.15 min; marked with *) with x-axis (retention time) and y-axis (absolute intensity). Experimental groups (A,B,C and D) and experimental group individuals are treated with *Lactobacillus* (E,F,G and H).

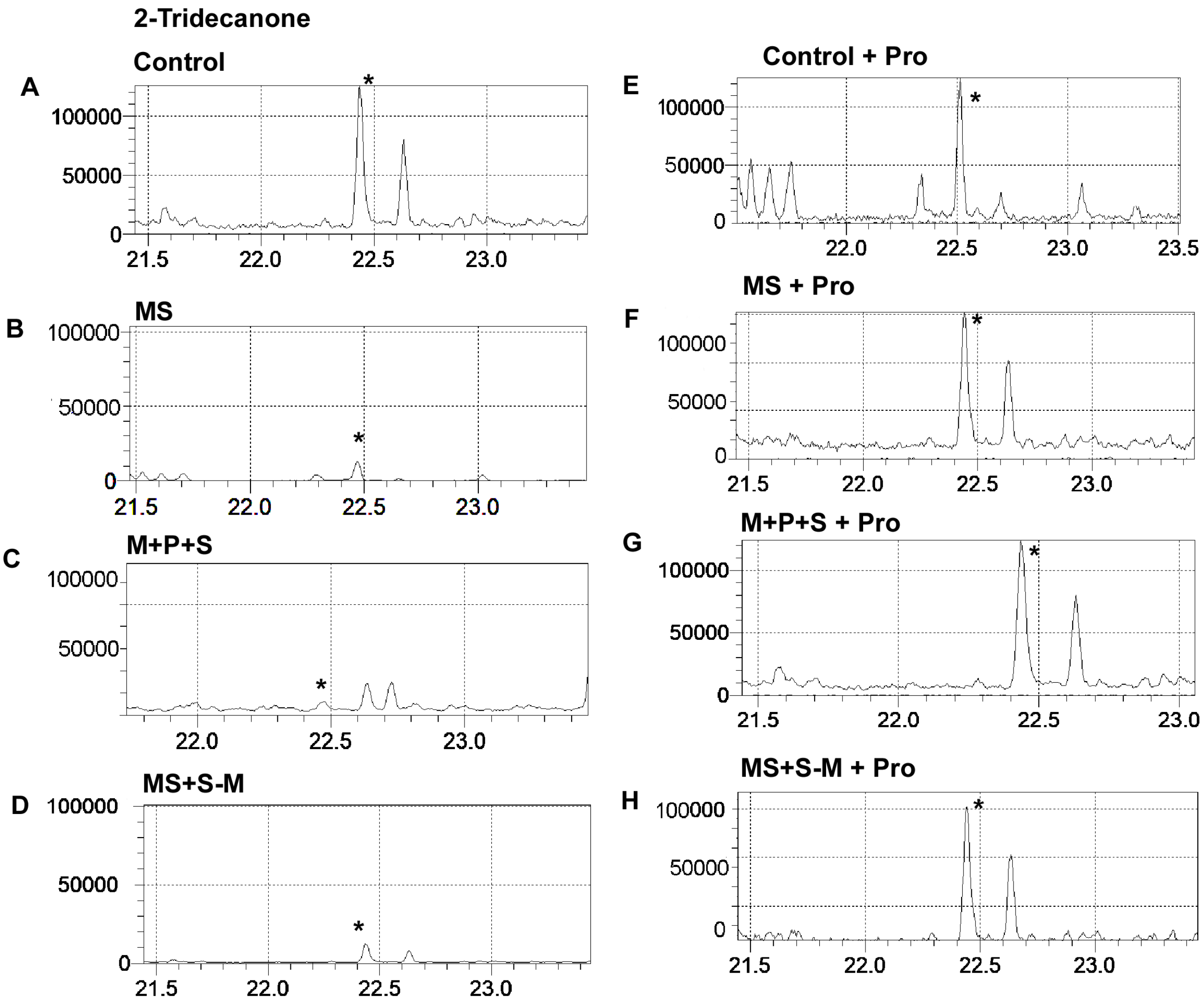

SUPPLEMENTARY FIGURE 12 GC-MS data for manuscript Figure 10E. Mass selective detector was run in scan mode to establish a fragmentation pattern of chromatogram and then using lab solutions GCMS postrun analysis software to get a specific peak (SIM chromatogram) of 2-Tridecanone (retention time – 22.46 min; marked with *) with x-axis (retention time) and y-axis (absolute intensity). Experimental groups (A,B,C and D) and experimental group individuals are treated with *Lactobacillus* (E,F,G and H).
